# Primary succession of microbial communities in an aquifer from the Covey Hill formation in Quebec, Canada

**DOI:** 10.3389/fmicb.2025.1568469

**Published:** 2025-05-21

**Authors:** Samuel Beauregard-Tousignant, Cassandre Sara Lazar

**Affiliations:** ^1^Biological Sciences Department, Université du Québec à Montréal (UQAM), Montreal, QC, Canada; ^2^Interuniversity Research Group in Limnology/Groupe de Recherche Interuniversitaire en Limnologie (GRIL), Montreal, QC, Canada

**Keywords:** continental subsurface, microbial communities, sessile microbes, microbial community succession, genomics

## Abstract

Aquifers in the continental subsurface have long been exploited for their resources. However, given the technical difficulties in accessing recurring subsurface samples, their community diversity and temporal dynamics remain largely misunderstood. Here, we investigated the effects of time and organic and inorganic carbon concentration variation on primary succession of microbial communities belonging to the Bacteria and Eukaryote domains colonizing rock surfaces and groundwater from a shallow fractured sandstone aquifer with a very high concentration of organic carbon and low concentration of nitrogen compounds. We attempted to recreate its physicochemical environment in a triplicate bioreactor setup and let the communities grow for 24 days. The sessile and planktonic communities were sampled daily in independent experiments and identified based on their 16S (Bacteria) or 18S (Eukaryote) rRNA genes. Time was the parameter with the strongest correlation both with alpha and beta diversity. The primary succession of all communities seems to have been divided into two temporal phases: in the first phase, approximately the two 1^st^ days, the variations in community composition and diversity were high. In the second phase, the variation is more progressive and lasted until the end of the experiment. As expected in an aquifer rich in organic carbon, bacteria were mostly heterotrophs, except in the first few days where there were some chemolithotrophs, and eukaryotes were heterotrophs or likely mixotrophs. Unexpectedly, the alpha diversity of the sessile and planktonic communities varied following similar patterns, but the planktonic ones varied with a wider amplitude. Regarding carbon's effect, organic and inorganic carbon concentration variation explained a much smaller proportion of the variation in alpha and beta diversity than expected. We believe this is due to its high concentration throughout the incubation and to the strong limiting effect of other factors such as nitrogen concentration and pH. The communities of both Bacteria and Eukaryotes were more active than expected and their temporal dynamics and interactions should be further investigated in varying carbon, nitrogen and other nutrient concentrations to better understand how different perturbations can affect subsurface communities and, subsequently, us.

## 1 Introduction

The continental subsurface provides us with countless services and resources, ranging from hydrocarbons and drinking water (Pearce et al., 2023; Smith et al., 2018) to potentially storing CO_2_ (Emerson et al., 2016) or H_2_ (Jin and Sengupta, 2024) to help mitigate climate change. Most if not all of these underground resources are influenced by subsurface microorganisms able to use, transform and degrade them (Jewell et al., 2016; Jin and Sengupta, 2024; Pearce et al., 2023). These communities also are essential to multiple biogeochemical cycles (Jewell et al., 2016; Kumar et al., 2017; Katayama et al., 2023) and remove nitrates from contaminated groundwater (Jakus et al., 2021). Thus, the impact of subsurface microbial activities may be high, but they are still poorly understood. They are comprised of all three domains of life (Bacteria, Archaea, and Eukaryote) (Rajala and Bomberg, 2023) and most often originate from surface water leaching into the subsurface in aquifer ecosystems (Yan et al., 2021). Once in the subsurface, these microorganisms can adopt one of two lifestyles: they can either be attached to a surface, in which case they are considered as sessile, or they can be free floating in (ground)water, in which case they are planktonic (Marshall, 2013). These sessile and planktonic communities, likely taxonomically distinct (Flynn et al., 2013), interact with each other (Rajala and Bomberg, 2023) and vary differently through time (Dong et al., 2021; Fillinger et al., 2018; Rajala and Bomberg, 2023) both in their metabolic activities and taxonomic composition (Dong et al., 2021). Predation and competition for resources between eukaryotes and bacteria are also known to take place (Herrmann et al., 2020), but are still poorly understood. While the sessile communities have been shown to be more active and faster growing than their planktonic counterparts (Griebler and Lueders, 2009; Sharma et al., 2024), the planktonic microbes seem to be more diverse (Patel et al., 2024; Sharma et al., 2024; Yan et al., 2020).

A few studies have observed the changes of subsurface community composition throughout the colonization of a sterile environment, also known as primary succession, in pristine aquifers (Sharma et al., 2024) or in flowback water from hydraulic fracturing wells (Hull et al., 2018). However, these studies sampled infrequently, looked at succession on a longer time scale and observed great changes occurring at every sampling point (Sharma et al. (2024) sampled after 0, 2, 4, 8 days and later; and Hull et al. (2018) sampled after 1, 4, 7 days and later). Some other studies took interest into the influence that outside events such as groundwater recharge had on the succession (Fillinger et al., 2021, 2018), but the effect of the time and of the perturbations often ends up being confounded. Most of these studies focused on bacteria and seldom studied the eukaryotic communities despite strong interactions such as predation and competition for resources taking place (Herrmann et al., 2020). In summary, the temporal dynamics of both bacteria and eukaryotes and the dynamics unique to the eukaryotes and the interactions between these two domains still are poorly understood despite being important.

The high variation observed in the early days of colonization may be explained by a multitude of factors including environmental ones. Indeed, the resources made available or used by microorganisms colonizing the environment (Fierer et al., 2010) and the competitive exclusion that may come from the activity of other microorganisms (Wetherington et al., 2022; Lee et al., 2023) could enable and favor different microorganisms to thrive through time. For example, some sessile prokaryotes are able to create biofilms, an extracellular polymer substance increasing the microorganisms' resistance to external stresses such as desiccation and nutrient deficiencies (Smith et al., 2018). This provides a strong competitive advantage to the microorganisms able to produce and live in it. The biofilm, however, is dependent on the microbial cell density; the community must reach a certain threshold density for the taxa to start forming a biofilm and that threshold varies between different taxa (Zhou et al., 2020). A wide variety of eukaryotes also thrive in the biofilm despite not being able to form one themselves, thus strongly influencing biofilm dynamics (Zirnstein et al., 2012).

While it is generally agreed that the development of all subsurface communities is partly stochastic (Fillinger et al., 2018), some factors relating to the community composition (Sharma et al., 2024) and the water geochemistry seem to play an important role (Fillinger et al., 2018) with parameters such as organic and inorganic carbon concentration and composition (Schwab et al., 2019), organic and inorganic electron donors (Herrmann et al., 2017), nitrogen compounds (Schwab et al., 2016), dissolved oxygen and the distance from the surface (Fillinger et al., 2018) playing a major role in the shaping of the community; potentially exercising more influence than time itself (Yan et al., 2020). The sessile community also seems to be strongly influenced by selection, but carbon availability is still expected to play an important role (Rajala and Bomberg, 2023).

If the concentration of organic carbon in an environment is low or non-existent, it is expected that autotrophs will be the first colonizers. Some chemolithotrophs can mineralize CO_2_ and play essential roles in nitrate reduction in oxic and anoxic environments (Kumar et al., 2017; Schwab et al., 2019; Visser et al., 2024). These slow growing early colonizers subsequently are preyed upon by metazoan top predators (Herrmann et al., 2020). Meanwhile, in an environment containing a high concentration of organic carbon, heterotrophs should play a bigger role in the early stages of succession (Fierer et al., 2010) and there is a negative correlation between fungi and chemolithotrophs (Herrmann et al., 2020). The carbon sources of a community and the interactions between prokaryotes and eukaryotes thus plays a crucial role in shaping subsurface communities.

In this study, we assessed the influence of time and dissolved organic and inorganic carbon concentration variation during the primary succession of sessile and planktonic microorganisms in a pristine fractured sandstone aquifer with a high carbon concentration and very low nitrogen concentration. Using amplicon sequencing targeting bacteria and eukaryotes, and a bioreactor setup, we followed succession of sessile microbial communities during the colonization of rock surfaces every 24 h for 24 days, as well as daily succession of the planktonic microbial communities in the water. This study is crucial in our understanding of the short-term community dynamics, the effect of different nutrient limitation and the interactions between these communities. When combined with further studies regarding the interactions of the different communities and the influence of varying nutrient concentration and of different contaminants on subsurface communities, it should enable us to better understand how aquifers may influence and respond to perturbations in the subsurface.

## 2 Material and methods

### 2.1 Experimental set-up

Given the near-impossible task of collecting subsurface rock samples daily to study microbial succession in an aquifer ecosystem, the *in situ* environment was recreated as closely as possible in a triplicate bioreactor setup (bioreactor 1, B1; bioreactor 2, B2; and bioreactor 3, B3).

Two different experiments were performed. One to examine sessile microbial colonization of rock surfaces from planktonic microbes and a second to investigate planktonic microbial colonization of groundwater from colonized rocks. In the first experiment (*E1*), the collected groundwater was circulated through the bioreactors and into collection bottles in an open system similar to an aquifer habitat where water only flows in one direction. The output water and a rock pellet were collected daily from each triplicate bioreactor for genomic analysis. Since the water was only collected once it had flowed through the bioreactor, the water collection did not disturb the incubation. The second experiment (*E2*) occurred in two phases. The first phase was conducted the same way as for the study of sessile succession, but for 21 days instead of 24 and without collecting any rock pellets to avoid disturbing the sessile community. The second phase then took place where first, the bioreactors were emptied of their water, only leaving the colonized rock pellets in the system. Then, autoclaved, freshly sampled groundwater was circulated for 24 days in the system to observe how the microorganisms that previously colonized the rock pellets would detach and settle in the groundwater. Three pellets from each bioreactor were collected twice: once when the bioreactors were emptied between that first and second phase and once on the last day of the second phase to assess how the community on the rocks had changed over time. It was decided to not collect rock pellets more frequently to avoid disturbing the water succession process as much as possible. A summary of the samples sequenced and analyzed in this study are presented in Table 1.

**Table 1:** Summary of the samples collected and processed for bacterial 16S and eukaryote 18S rRNA gene sequencing, and analyses.

**Experiment**	**Rate of sampling**	**Bioreactor 1**	**Bioreactor 2**	**Bioreactor 3**
**E1**	Rock and water samples collected daily for 24 days	−24 rock	−24 rock	−24 rock
		−24 water	−24 water	−24 water
**E2 Phase 1**	Water emptied from bioreactors after 21 days Rock pellets are left in place			
**E2**	Water samples collected daily for 24 days	−24 water	−24 water	−24 water
**Phase 2**	Rock samples (3) collected at Day 1 and Day 24	−6 rock	−6 rock	−6 rock

B, bioreactor; E, experiment.

### 2.2 Study site and sampling

Groundwater was sampled during the summer of 2022 from a 1.5 m deep well using a capillary pump (Waterra D-25 Foot Valve, Canada) near Covey Hill, in the province of Quebec, Canada (45°00′27.6^′′^N 73°49′10.5^′′^W). The well was first purged to get rid of stagnant water by pumping until the water's physico-chemical parameters, measured using a multi-parameter probe (YSI 650 MDS, USA), were stabilized (approximately 30L of purged water). The geochemical and physical properties were measured *in situ* (Table 2). The aquifer is composed of fractured sandstone and has an estimated flow rate of 2 × 10^−5^ to 4 × 10^−5^ m/s (Nastev et al., 2008). Despite limited study, further characterization of the aquifer can be obtained from the literature (Levison et al., 2013, Nastev et al., 2008). Following the purge, 1L and 10L of groundwater were collected in alternance until four 1L sterile polypropylene bottles (Nalgene, Sigma-Aldrich, St. Louis, MO, USA) and three 10L opaque, sterile PYREX low-actinic glass bottles were filled. The 1L bottles enabled us to establish the initial *in situ* groundwater microbial community diversity and composition and the 10L bottles were used for the bioreactor incubations. Since the geological formation at 1.5 meters is the same for the surface rocks (Girard et al., 2015), we sampled rocks from the surface to be used in the bioreactors. Because the aquifer is recharged from a nearby peat bog (Levison et al., 2013), 1L of water was also collected from its surface to assess the influence of that recharge site on the aquifer communities. The experimental set-up and flow are the same as described in Patel et al. (2024). A visual summary of the experimental set-up can be found in Supplementary Figure 1.

**Table 2:** Raw geochemical measurements for all field samples.

**Sample type**	**Temperature (°C)**	**pH**	**Dissolved O_2_ (%)**	**DIC (mg/L)**	**DOC (mg/L)**	**Conductivity (μS/cm)**	**NO2 (mg/L)**	**NO3 (mg/L)**	**NHx (mg/L)**
Peatbog *E1*	19.04	4.04	91.2			22			
Groundwater *E1*	13.55	4.73	80.9	13.41	29.21	24	0.01	0.0015	0.08
Peatbog *E2*	20.41	3.83	61.4	11.8	39.4	17			
Groundwater *E2*	18.34	4.42	61.6	15.6	25.85	28	0.02	0.0005	0.08

### 2.3 Water geochemical analyses

To determine the dissolved inorganic and organic carbon concentration (DIC and DOC) of the water in the field and during the experiments, samples were taken and filtered on 0.45 μm filters (Sarstedt^®^, Numbrecht, Germany) into gas-free glass bottles. These bottles were stored at 4°C and analyzed with an OI Analytical Aurora 1030W TOC Analyzer (https://www.oico.com/1030W) using a persulfate oxidation method at the GRIL-Université du Québec à Montréal (UQAM) analytical laboratory.

Regarding nitrogen compounds, to determine ammonium and ammonia (NH_x_) concentrations, water samples were collected in plastic scintillation vials after filtration on a 0.2 μm polyether sulfone filter (Sarstedt^®^, Numbrecht, Germany). Samples were analyzed on a Flow Solution 3100 autosampler using a chloramine reaction with salicylate to form indophenol blue dye (EPA Method 350.1). For measurement of nitrates (NO_3_) and nitrites (NO_2_), water samples were collected in plastic scintillation vials after filtration on a 0.45 μm polyethersulfone filter (Sarstedt^®^, Numbrecht, Germany). Samples were analyzed with a continuous flow analyzer (OI Analytical Flow Solution 3100©, OI Analytical, College Station, TX, USA) using an alkaline persulfate digestion method, coupled with a cadmium reactor, following a standard protocol (Patton and Kryskalla, 2003) at the GRIL-Université du Québec à Montréal (UQAM) analytical laboratory.

### 2.4 Rock pellets preparation

To prepare the rock slabs collected in the field and create the pellets installed into the bioreactors, the slabs were first cleaned with a brush, soap and water to remove any dirt and vegetation. Core plugs were then drilled into the slabs using a 5/8 inches diamond hole saw (Milwaukee, 49-56-0513, Taiwan) fixed on a drill press (18′' nova voyager dvr, King Industrial, Canada). These plugs were then cut approx. 0.3 cm thick and ground to fit into the bioreactor's slots using a rotary tool (DREMEL 3000, DREMEL, Mount Prospect, IL, USA). The pellets obtained were further cleaned by vortexing them in milli-Q water and detergent, cleaning them and finally, sonicating (Sonifier Cell Disruptor 185, Branson, Rungis, France) them for 1 min. These pellets were then air dried and autoclaved before being installed into the bioreactor. The whole bioreactor setup with the tubing and the pellets were autoclaved prior to incubation experiments.

### 2.5 Bioreactor set-up

The bioreactors (CBR 90 Standard CDC Biofilm Reactor, BioSurface Technologies, Bozeman, MT, USA) were continuously agitated with a magnetic stirrer. They also were wrapped in aluminum foil and placed in a dark incubation chamber in a windowless basement to minimize as much as possible light exposure. Using a peristaltic pump (IPC, Ismatec, Malente, Germany), groundwater was pumped through these bioreactors at a rate of 0.289 ml/min. The bioreactors contained 8 columns of 3 pellets each for a total of 24 pellets per replicate. The bioreactors were supplied with 200 sccm of a varying mix of gasses (N_2_, O_2_, and CO_2_) using a gas mixer MCQ GB100 (Monkey Industrial Supply, USA). This mix was calculated based on dissolved oxygen and pH measured in the groundwater on the day of sampling and the temperature of the growth chambers was set at the temperature measured in the field (Table 2). The dissolved O_2_ and pH in the water were measured daily by collecting 50 ml of water from each bioreactor and using a probe (accumet XL600, Fisher Scientific, USA). These readings were then used to adjust the gas flow to the bioreactors to keep the abiotic conditions as close as possible to the ones measured in the groundwater extracted from the aquifer on the day of sampling.

A blank run was performed for 21 days using the same set-up previously described but using sterile milli-Q water and rock pellets prepared as the previous ones but also dipped 3 times in HCl 10% and then rinsed using de-ionized water before being autoclaved. DNA was extracted from one of the rock pellets obtained from each bioreactor, to be considered as contamination and removed from further analysis.

The water that flowed out of the bioreactors was filtered on sterile polyether sulfone membrane filters with 0.2 μm diameter pores (Sartorius, Göttingen, Germany) using a vacuum pump (Welch 2019B-01, Welch, USA). They were then kept at −80°C until extraction. Similarly, the pellets collected daily were put into sterile tubes and stored at −20°C until extraction.

### 2.6 Groundwater sterilization for the second phase of the second experiment

We first tried sterilizing the groundwater in 1L clear PYREX bottles using a UV-clave (Benchmark, U.S.A), but it failed to reduce DNA concentrations below the detection threshold of the Qubit^TM^. It then was decided to autoclave the groundwater, and this successfully reduced DNA concentration below detection thresholds. It was, however, observed that flakes formed in the water following the autoclave. Blanks of the sterilized water were also sequenced and removed from the subsequent dataset.

### 2.7 DNA extraction and sequencing

DNA was extracted from the water filters using the DNeasy PowerWater kit (QIAGEN, Germany). The DNA from the sessile community (the rock pellets) was extracted using the protocol detailed in Patel et al. (2024). To summarize, the rock pellet-attached microbial cells were first scrapped with zirconium sand and the cells were lysed using SDS 20%. The DNA was then isolated using 25:24:1 phenol:chloroform:isoamyl alcohol, precipitated using ethanol, sodium acetate and glycogen, air dried and resuspended in TE buffer. The extracted DNA was stored at −20°C until further processing. Blank kit extractions were performed, and the sequences obtained from these runs were removed from analysis. The amplification of the community of B1, day 21 of the first experiment did not work so this sample has been removed from further analyses.

Despite our best efforts, the archaeal domain could not be reliably amplified and thus, was not analyzed. Similar observations were made in Patel et al. (2024). This may be due to their low relative abundance when compared to bacteria and lower number of gene copies per cell (Hoshino and Inagaki, 2018; Opitz et al., 2014) potentially putting them below our detection threshold. There also may have been a mismatch of primers making the PCR amplification less effective and the co-extraction of humic acids may have hindered the PCR reactions (Hoshino and Inagaki, 2018). These four factors combined pose some great challenges to PCR amplification and, especially, to archaeal amplification. The effect of these factors may be particularly strong during the early stages of primary succession where the absolute abundance of microorganisms must have been extremely low.

The primer pairs B341F–B785R (5′-CCTACGGGAGGCAGCAG-3′ and 5′-GACTACHVGGGTATCTAATCC-3′) was used for the Bacteria domain, E960F–E1438R (5′-GGCTTAATTTGACTCAACRCG-3′ and 5′-GGGCATCACAGACCTGTTAT-3′) for the Eukaryote, and A340F-A915R (5′-CCC TAH GGG GYG CAS CA-3′ and 5′-GTG CTC CCC CGC CAA TTC CT-3′) for the Archaea (the amplification conditions can be found in the Supplementary Table 1). Samples were sent to the CERMO-FC (Center of Excellence in Research on Orphan Diseases – Fondation Courtois) at UQAM to be sequenced using Miseq Illumina and a Miseq Reagent v3 600-cycle kit (Illumina, San Diego, CA, USA) with a paired reading of 300 bp. Before sequencing, Phix control library (Illumina) was spiked into the amplicon pool to improve the unbalanced base composition. Negative PCR controls and the control samples for water and rock samples were sequenced for the two domains. The raw reads were deposited into the National Center for Biotechnology Information (NCBI) under the BioProject ID (PRJNA1159903).

### 2.8 Bioinformatic and statistical analyses

For all analysis, the R software (v4.2.2; R Core Team, 2024) was used. The DADA2 package (v1.26.0; Callahan et al., 2016) was used to process the raw reads and produce amplicon sequence variant (ASV) tables. Sequences present in the controls were considered contaminants and were removed from the datasets using the decontam package (v1.24.0; Davis et al., 2018). For both domains (Bacteria and Eukaryote), rarefaction was carried out using the median sequencing depth method. Taxonomy was assigned using the Silva database v.138.1 for the prokaryotes (Glöckner et al., 2017; Quast et al., 2012; Yilmaz et al., 2013), and the PR2 database for the eukaryotes (del Campo et al., 2018; Guillou et al., 2012; Vaulot et al., 2021, 2022). The ggplot2 package (v3.5.1; Wickham, 2016) was used for data visualization. A significance threshold of 0.05 was used for all experiments. The environmental parameters (DIC and DOC concentrations, pH, percentage of dissolved oxygen, time, and bioreactors) were tested for autocorrelation using the ggpairs function with a Pearson correlation coefficient. If the correlation exceeded the 0.7 threshold, only one of the parameters was conserved. The pH and the percentage of dissolved oxygen had high correlations with multiple parameters of both experiments and thus, were excluded and, since DOC and time were strongly colinear for the second experiment *E2*, DOC was excluded from that analysis.

The community was first visualized by stacked bar chart. The proportion of shared ASVs between the sessile and planktonic communities on each day for *E1*, and the unique share of ASV for each lifestyle (planktonic or sessile) was further established to determine the temporal behavior of each community.

Since the Shannon index can be influenced either by richness or evenness of the community, alpha diversity, richness and evenness indices were computed using the estimate_richness function of the Phyloseq package (v1.48.0; Mcmurdie and Holmes, 2013). The alpha diversity index used was the Shannon index. The richness was the observed richness, selected since it is a component of the Shannon index. The evenness was Pielou's index, calculated by dividing the Shannon index by the natural logarithm of the observed richness. Thus, by comparing the variations of richness and evenness, we could have a direct explanation of the variations of Shannon index. The variations in all these measures were analyzed using the vegan package (v2.6-6.1; Dixon, 2003). Finally, a time series analysis of the variation of Shannon index through time, the variations of DIC and DOC concentrations and the variations between bioreactors was performed. To do so, hierarchical generalized additive models (HGAM) were done by using the mgcv package (v1.9-1; Wood, 2010) as the data was not expected to be normal and we also wanted to factor in the influence of parameters that were expected to have an unknown, but most likely non-linear influence on the variable of interest. We also desired to include hierarchical effects to account for the fact that each bioreactor inevitably had minute differences (e.g., pellet composition, differences in the initial community) that could influence the outcome of the experiment, especially since a part of the community variation is expected to be stochastic. DIC and DOC concentration variations were suspected of having a great influence on alpha diversity. Since the relationship and linearity of their influence was unknown, we decided to use an anisotropic tensor function including DIC and DOC concentration and their interaction for the modelization of the sessile community. However, since DOC concentration and time were highly correlated in the second experiment, it was removed from the modelization of *E2*. Time and the individual bioreactors were also suspected of having a strong influence on Shannon indices, so time was included as a main effect and the variation of each bioreactor from the main effect was included to enable us to determine a general trend and the unique behavior of each bioreactor. A random effect for the bioreactors was also included to accommodate the varying initial alpha diversity of each bioreactor. The Gamma family with an identity link function was used as the data is strictly positive and not necessarily normal. A k-check was performed and the qq-plot, residuals vs. fitted, and histogram of residuals were visually inspected to determine the validity of the model and the potential biases. We set the hyperparameter m = 1 for the group trends (Pedersen et al., 2019). The models were constructed on this framework:

Shannon ~ te(dic, doc) + s(time) + s(time, by = bioreactor, m = 1) + s(bioreactor, bs = “re”)

For beta diversity analyses, visualization of the data was computed using NMDS ordination and clustering (20 iterations), with the ordinate function of the Phyloseq package. A db-RDA (capscale function, vegan package) and a subsequent one degree of freedom sequential contrast ANOVA (999 permutations, anova.cca function, vegan package) were performed on both domains to determine if the lifestyle, the experiment and the bioreactors were significantly different from one another. We then carried out a db-RDA (capscale function, vegan package), a one degree of freedom sequential contrast ANOVA (999 permutations, anova.cca function, vegan package) and variance partitioning (varpart function, vegan package) to determine which proportion of the variance in the beta diversity could be attributed to the different geochemical parameters and if the bioreactors behaved in similar ways through time. Given the nature of contrast tests, this test only allowed us to compare B2 and B3 to B1. In the case where both B2 and B3 were not significantly different from B1, a *post-hoc* test would have been performed to compare B2 and B3 to determine if all three bioreactors were not significantly different. It however never was the case.

Finally, the origin (or contribution from previous time point communities) of each community was determined using the FEAST algorithm for microbial source tracking of the FEAST package (v0.1.0; Shenhav et al., 2019). The community of the same lifestyle from the previous days, the community from the other lifestyle, the groundwater sampled in the field and the peat bog water from the sampling day were used to determine how much of the community of a single day could be explained by each of these different sources.

During the first incubation (*E1*), the measures of the physico-chemical parameters of the 2^nd^ day were used for the first day as well for the alpha and beta diversity variation analysis in relation to geochemical parameters since, during the incubation, we were not able to retrieve enough water to measure them on the 1^st^ day.

## 3 Results

### 3.1 Water physico- and geo-chemical features

The bioreactor set-up was not able to accurately reproduce the *in situ* pH and dissolved oxygen percentage despite all of the pumped gas being either CO_2_ (to decrease pH) or O_2_ (to increase the percentage in dissolved oxygen). The recreated conditions however were all well within normal variation observed in the aquifer during the summer of the sampling (2022). The measurements taken *in situ* reveal that the aquifer was oxic (dissolved O_2_ *E1*: 80.9 %, *E2*: 61.6 %), acid (pH *E1*: 4.73, *E2*: 4.42), and had high concentrations in DIC (*E1*: 13.41 mg/L, *E2*: 15.6 mg/L) and DOC (*E1*: 29.21 mg/L, *E2*: 25.85 mg/L). Its conductivity was low (*E1*: 24 μS/cm, *E2*: 28 μS/cm) and its nitrogen content was very low (*E1:* NO_2_: 0.01 mg/L, NO_3_: 0.0015 mg/L, NH_x_: 0.08 mg/L, E2: NO_2_: 0.02 mg/L, NO_3_: 0.0005 mg/L, NH_x_: 0.08 mg/L). In the first experiment, pH remained mostly below 6 (Mean: 5.03) whereas in *E2*, pH remained below 5 (Mean: 4.41). Both experiments were oxic with a mean percentage of dissolved O_2_ of 55.34% for *E1* and of 59.70% for *E2* (Supplementary Table 2). DIC concentration (Mean *E1*: 41.78 mg/L, Mean *E2*: 48.93) (Supplementary Table 2) had generally higher values in B1 than B2 and B3 in *E1* (Supplementary Figure 2). DOC concentrations remained rather similar for all three bioreactors for both experiments (Supplementary Figure 2) and had a mean concentration of 29.17 mg/L for *E1* and of 19.16 mg/L for *E2* (Supplementary Table 2).

Physico- and geo-chemical parameters remained largely constant in-between bioreactors except for DIC concentration in *E1* (daily primary succession of sessile microbial communities on rock surfaces) (Supplementary Figure 2). During *E1*, pH was correlated with DIC concentration and time (Pearson correlation coefficient: 0.613 and 0.542) and the percentage of dissolved oxygen was correlated with DIC and DOC concentration, time and pH (0.361, 0.328, 0.657, and 0.679). During *E2* (primary succession of planktonic communities in groundwater), DOC concentration was strongly correlated with time, and pH was correlated with DIC concentration (0.895 and 0.807) (Supplementary Figure 2). Given their high collinearity with multiple parameters, pH and dissolved oxygen concentration were removed from the analysis and DOC concentration was removed from the analysis of the second experiment to avoid collinearity problems with time.

### 3.2 Community taxonomic composition

**Figure 1:**
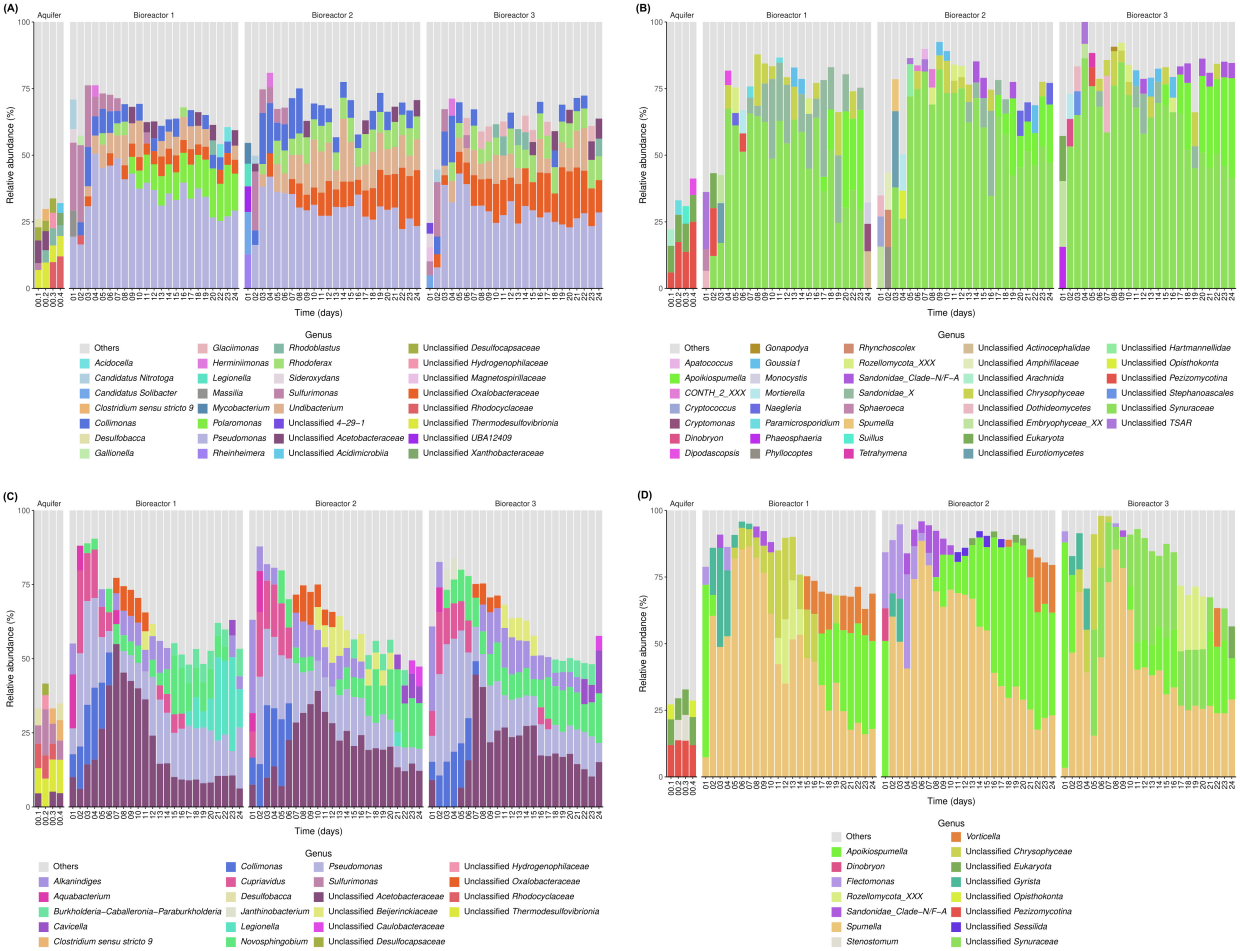
Taxonomical identification of each community based on 16S/18S rRNA gene sequencing: **(A)** sessile Bacteria of *E1*; **(B)** sessile Eukaryotes of *E1*; **(C)** planktonic Bacteria of *E2*; and **(D)** planktonic Eukaryotes of *E2*.

Overall, throughout the incubations, 13,067 bacterial ASVs and 3,467 eukaryotic ASVs were observed. For experiment *E1*, for the Bacteria domain, the initial *in situ* groundwater had a high diversity, and no genera occupied more than 10% of these communities (Figure 1A). Unclassified (unc.) *Acetobacteraceae* and unc. *Thermodesulfovibrionia* were the most abundant genera for the first liter recovered before the first 10 L were pumped (00.1) and the second liter collected after the first 10L was pumped (00.2). The third pumped liter (after the second 10L) saw a gradual change in the *in situ* groundwater bacterial community (00.3) where unc. *Rhodocyclaceae, Rhodoblastus*, unc. *Thermodesulfovibrionia* and unc. *Hydrogenophilaceae* were most abundant, while the last liter collected after all the groundwater for the incubation experiments was recovered (00.4) saw the introduction of unc. *Xanthobacteraceae* in the main genera. For the eukaryotes, the initial *in situ* groundwater sample 00.1 contained unc. *Eukaryota*, unc. *Pezizomycotina* fungi, and unc. *Arachnida*, while sample 00.2 contained unc. *Pezizomycotina*, unc. *Eukaryota* and *Paramicrosporidium*; and samples 00.3 and 00.4 had unc. *Pezizomycotina*, unc. *Eukaryota* and the fungi, *dipodascopsis* and *Suillus* (Figure 1B).

Regarding the Bacteria domain of *E1* for the sessile community, the first 2 days showed the most variation and differences compared to the following days within each bioreactor, but also between them (Figure 1A). The first day for B1 was dominated by *Sulfurimonas, Pseudomonas*, and candidatus (cand.) *Nitrotoga* while B2 was dominated by cand. *Solibacter, Rheinheimera*, and unc. UBA12409 (*Nitrospirota* phylum) and B3 by *Sulfurimonas*, unc. *Magnetospirillaceae*, and *Sideroxydans*. On the 2^nd^ day all three bioreactors were dominated by *Sulfurimonas, Pseudomonas*, and *Collimonas*, and for all bioreactors, *Pseudomonas* dominated the following days. For bioreactor B1, the same 3 genera dominated until day 5, where *Undibacterium* was more abundant than *Sulfurimonas*. On day 9, *Polaromonas* was more abundant than *Collimonas*, and slowly increased until the end of the incubations. For bioreactors 2 and 3, *Collimonas* was another major genus for days 4 and 5. After day 5, alongside *Pseudomonas*, unc. *Oxalobacteraceae, Undibacterium*, and *Rhodoferax* dominated.

In the planktonic bacterial community of *E1*, the community coming from the groundwater directly, a succession of heterotrophs can be observed, but there also is the presence of a chemolithotroph, *Sulfurimonas*, all throughout the incubation. After the 1^st^ day where it had a high relative abundance, it stayed at a constant abundance of <10% for the remainder of the incubation. There also was the presence of a photosynthetic bacteria, *Rhodoblastus* (Spring et al., 2013), from day 8 onwards (Supplementary Figure 3A).

For *E1*, for the sessile community of the Eukaryote domain, the first few days (2 to 4 depending on the bioreactor) had a very high diversity and changed rapidly (Figure 1B). The 1^st^ day for B1, was dominated by unc. TSAR, unc. *Dothideomycetes*, and *Sphaeroeca*, while days 2 and 3 contained many unc. *Embryophyceae*. The 1^st^ day for B2 and B3 was dominated by unc. *Embryophyceae* and unc. *Dothideomycetes*. After 5 days of incubation, for all bioreactors, unc. *Synuracea* and unc. *Chrysophyceae* became the most important genera. *Sandonidae*_X became more abundant around day 10 followed by *Apoikiospumella* around day 16. The samples collected in the field differed from the samples from the 1^st^ days for both domains, containing a much broader diversity that did not appear in a similar way in the incubation afterwards.

In *E1*, regarding the planktonic community used to colonize the sessile community, accurate identification could not be achieved for most ASV, but there was the presence of potentially mixotrophic protists (*Apoikiospumella*, unc. *Chrysophyceae* and unc. *Synuraceae*) as well fungi (*Rozellomycota*_XXX and *Pezizomycotina*) and *Goussia1*, a parasite (Jowers et al., 2023) (Supplementary Figure 3B).

For the Bacteria domain of *E2*, the main genera present in the sessile communities colonizing the sterile water were *Rhodoferax*, unc. *Acetobacteraceae*, unc. *Oxalobacteraceae*, and *Undibacterium* (Supplementary Figure 4A). Regarding the aquifer, no genera occupied more than 10% of the initial communities, but the main ones were unc. *Acetobacteraceae*, unc. *Thermodesulfovibrionia*, unc. *Rhodocyclaceae, Sulfurimonas*, and *Desulfobacca* for the first liter recovered before the first 10 L were pumped (00.1); *Sulfurimonas*, unc. *Thermodesulfovibrionia*, unc. *Rhodocyclaceae*, unc. *Desulfocapsaceae* and unc. *Hydrogenophilaceae* for 00.2; unc. *Thermodesulfovibrionia, Sulfurimonas*, unc. *Rhodocyclaceae*, and *Clostridium* for 00.3; and unc. *Thermodesulfovibrionia, Clostridium, Sulfurimonas*, and *Desulfobacca* for 00.4 (Figure 1C).

For the planktonic community of the Bacteria domain for *E2*, the first 2 days showed the most variation and differences compared to the following days within each bioreactor (Figure 1C). The 1^st^ day of incubation was dominated by *Aquabacterium, Alkanindiges*, unc. *Acetobacteraceae, Pseudomonas, Collimonas*, and *Cupriavidus*. The 2^nd^ day of incubation, all three bioreactors were dominated by *Pseudomonas, Cupriavidus, Collimonas, Alkanindiges*, and *Aquabacterium*. *Pseudomonas, Collimonas*, and *Cupriavidus* also dominated the bioreactors for days 4 and 5 (B1 and B3) and days 4, 5 and 6 for B2. Then, unc. *Acetobacteraceae* took a predominant role and increased until days 7 to 10 and then varied in relative abundance until the end of the incubations. *Novosphingobium* and *Pseudomonas*, together with unc. *Acetobacteraceae* were then the most important genera until the end.

For the eukaryotes of *E2*, the most abundant genera of the initial *in situ* groundwater were unc. *Pezizomycotina* fungi, unc. *Eukaryota* and unc. *Opisthokonta* for the first liter recovered before the first 10 L were pumped (00.1) and the last (00.4) and for 00.2 and 00.3, unc. *Pezizomycotina*, unc. *Eukaryota* and *Stenostomum* were the most abundant, still not having more than ca. 10% of relative abundance (Figure 1D). The rock pellets of *E2* were colonized at more than 75% by *Apoikiospumella* algae, but this colonization markedly decreased on the 24th day (Supplementary Figure 4B).

For the planktonic eukaryotic community of *E2, Apoikiospumella* was the most abundant genera during the 1^st^ days of the incubations (Figure 1D). Then *Spumella* dominated until the days 15–16. Afterwards, *Apoikiospumella* increased again in relative abundance. *Sandonidae*_Clade-N/F-A were important during the first 7–8 days for B1 and B2. Unc. *Chrysophyceae* were abundant during the middle of the incubations for B1 and the unc. *Synuraceae* for B3. Finally, *Vorticella* increased at the end of the incubations after days 13 for B1 and 20 for B2 and B3.

### 3.3 Shared ASVs between sessile and planktonic communities

**Figure 2:**
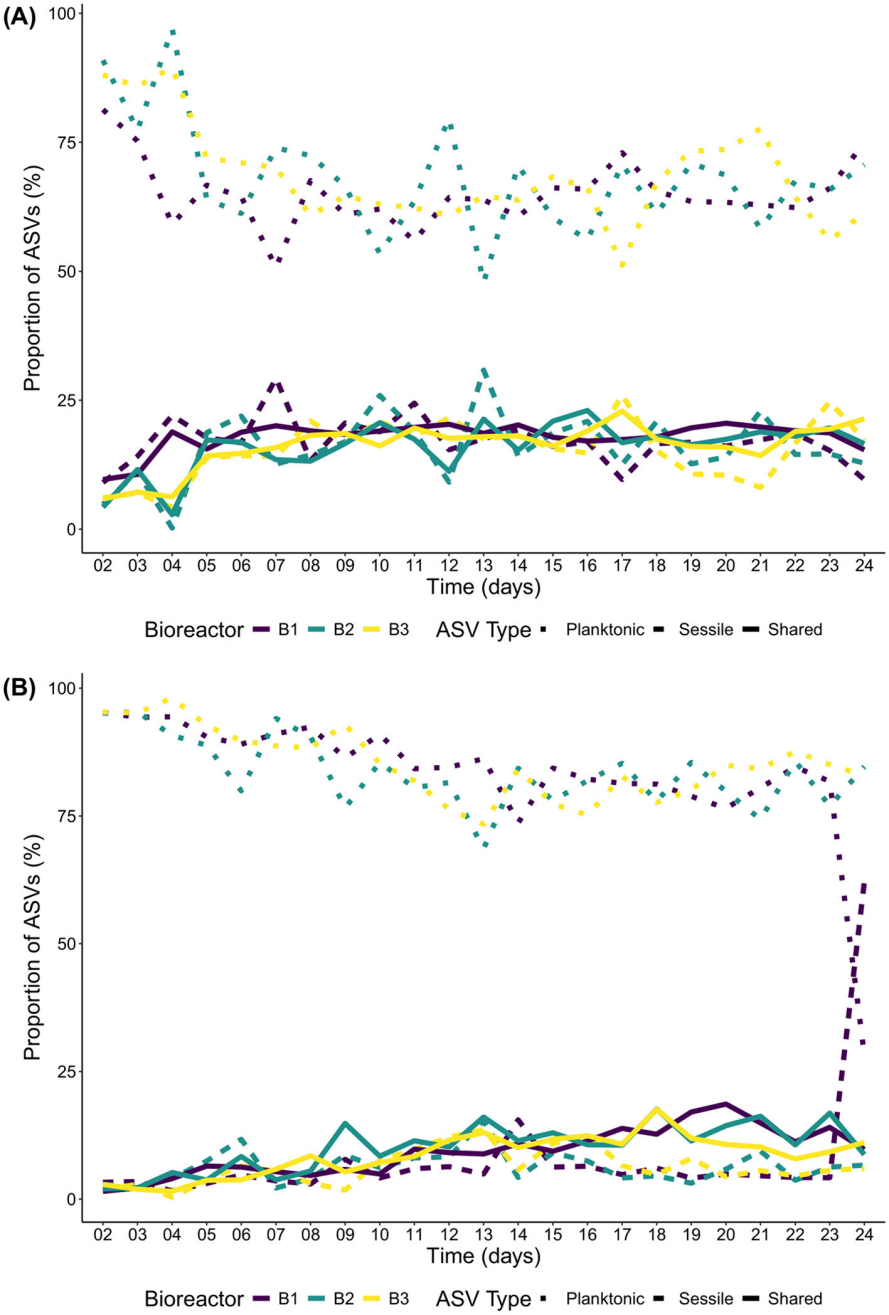
Shared and unique ASV percentage through time between the sessile and planktonic communities of *E1*. **(A)** Bacteria; and **(B)** Eukaryotes.

For the Bacteria domain of *E1*, the percentage of shared ASVs between the sessile and planktonic communities on the 2^nd^ day of incubations started between 5 and 12% and increased to reach a plateau at 20% to 25% after 5 days of incubation (Figure 2A). For the Eukaryote domain, the percentage of shared ASVs started at 2.5% and increased to reach a peak of 15% after 19 days of incubation and slightly decreased afterwards until the end of the incubations to end around 10% on day 24 except for B1 that, on day 24, had more than 50% of its ASVs unique to the sessile community (Figure 2B). Most of the unique ASVs were found in the planktonic communities of both domains, close to 90% on the 1^st^ day of the incubations and decreased to reach a plateau of 60% to 75% on day 6 for the Bacteria and day 15 for the eukaryotes (except for B1 which behaved differently on day 24). The unique ASVs present in the sessile communities were close to zero on the first day and remained at 25% or less for the rest of the incubation days for the Bacteria, and at 15% or less for the eukaryotes. Since the sessile communities used to colonize planktonic communities were only sampled on the first and last days of *E2*, the information is limited to only 2 timepoints and will not be discussed further.

### 3.4 Alpha-diversity variation and modeling

The alpha-diversity values (Shannon index) ranged, for the incubation, from 2.302 to 4.370 for the sessile bacteria of *E1*, from 2.766 to 4.514 for the planktonic bacteria of *E2*, from 1.189 to 4.252 for the sessile eukaryotes of *E1* and from 0.9521 to 3.0671 for the planktonic eukaryotes of *E2* (Supplementary Figure 5). The Shannon index of the sessile bacterial communities of *E1* started fairly low (<3, except for B3 at around 4), increased on day 2 to more than 4 and then decreased sharply on day 3 before stabilizing and slowly increasing for the remainder of the experiment. The planktonic bacteria of *E2* followed a rather similar trend, but the highest index of the first few days, close to 4, was on day one, followed by a sharp drop on day 2 and a subsequent increase for the remainder of the experiment with a plateau of around 4.25 starting around day 17. Regarding the eukaryotes, the sessile communities of *E1* started high as well (between 2.5 and 4) but decreased until close to day 7 to be around 1.5 before increasing slowly to be around 2 on the last day. The planktonic eukaryotes of *E2* were around 2 on the 1^st^ day (except B3). A subsequent decrease to 1 followed until ca. day 7 followed by a strong increase to end between 2 and 3.

The Shannon index of planktonic and sessile communities was relatively high, and the initial evenness of the sessile communities was high (Supplementary Figure 6) whereas the initial richness of the planktonic communities was high (Supplementary Figure 7). Following these first few days of high variation, both richness and evenness followed broadly similar trends to the Shannon index.

The richness and the Shannon index also were generally higher in the *in situ* samples than during the incubation period. The eukaryotes, except for the sessile community of *E1*, had a much broader variation between the four samples collected *in situ* than the bacteria (Supplementary Figures 5, 7). The evenness of both domains for the sessile experiments of *E1* was close to the evenness of the early days of the incubation, but the evenness of the *in situ* samples of *E2* were generally higher than what was observed during the incubation (Supplementary Figure 6).

**Figure 3:**
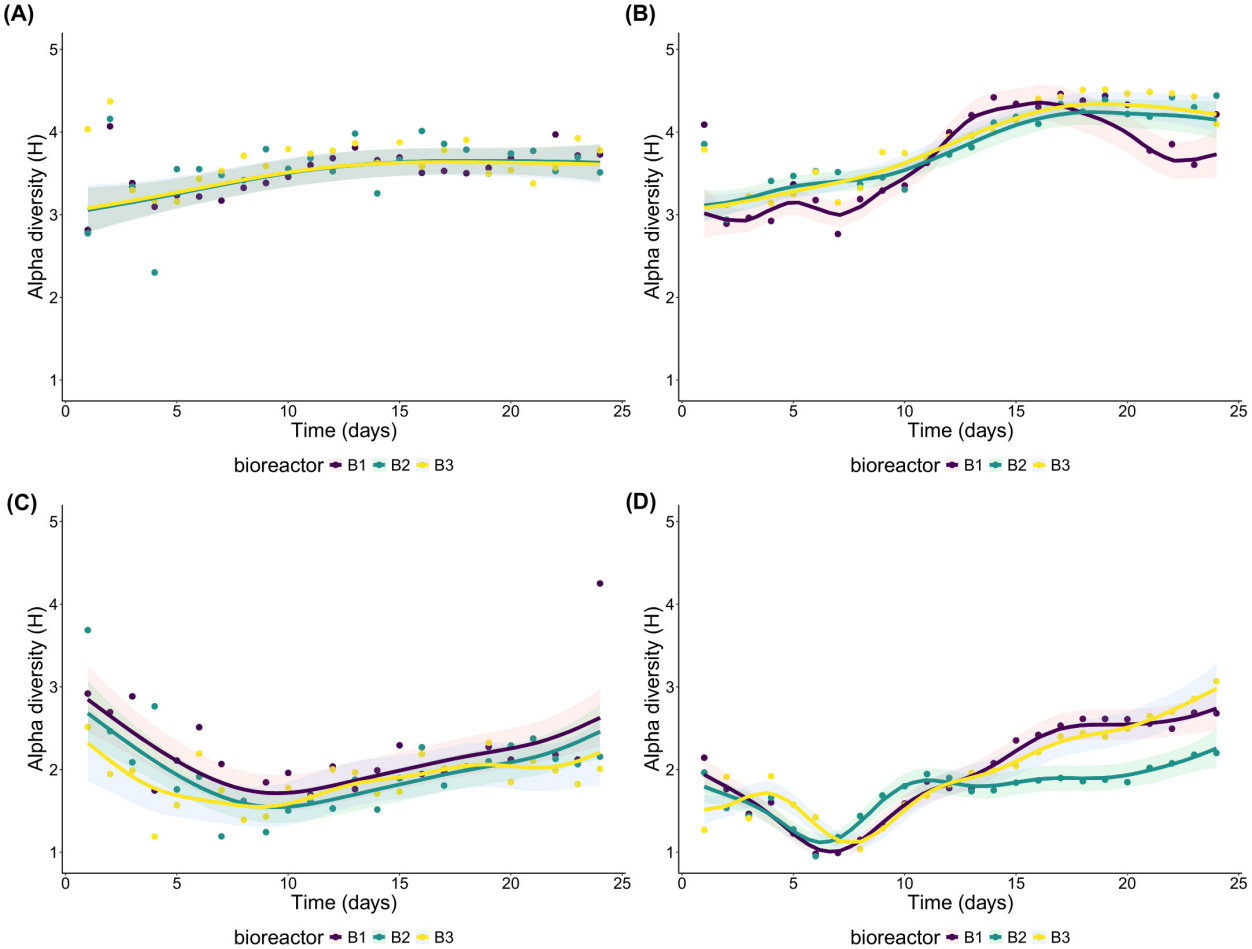
Combined effect of the shared trend of time and the individual effect of each bioreactor on the alpha diversity. **(A)** sessile bacteria of *E1*; **(B)** planktonic bacteria of *E2*; **(C)** sessile eukaryotes of *E1*; and **(D)** planktonic eukaryotes of *E2*.

Overall, the models aiming to attribute the source of variations of Shannon index for each community and each domain were able to explain much more of the variation in alpha diversity for the primary succession of the planktonic communities (*E2*) than the sessile one (*E1*) (Supplementary Table 3). The models for both lifestyles of the bacterial communities, however, were unable to accommodate the much higher diversity measured during the first 2 days for the sessile community (especially the 2^nd^ day) and during the 1^st^ day for the planktonic community (Figures 3A, B). The Shannon index for these days were ca. 4, but the models fitted an index closer to three. These divergences, along with day 24 of B1 of the sessile eukaryotes of *E1* (Figure 3C) were the only major divergences between the data and the model since the modelization of the planktonic eukaryotes of *E2* was fairly successful (Figure 3D).

**Figure 4:**
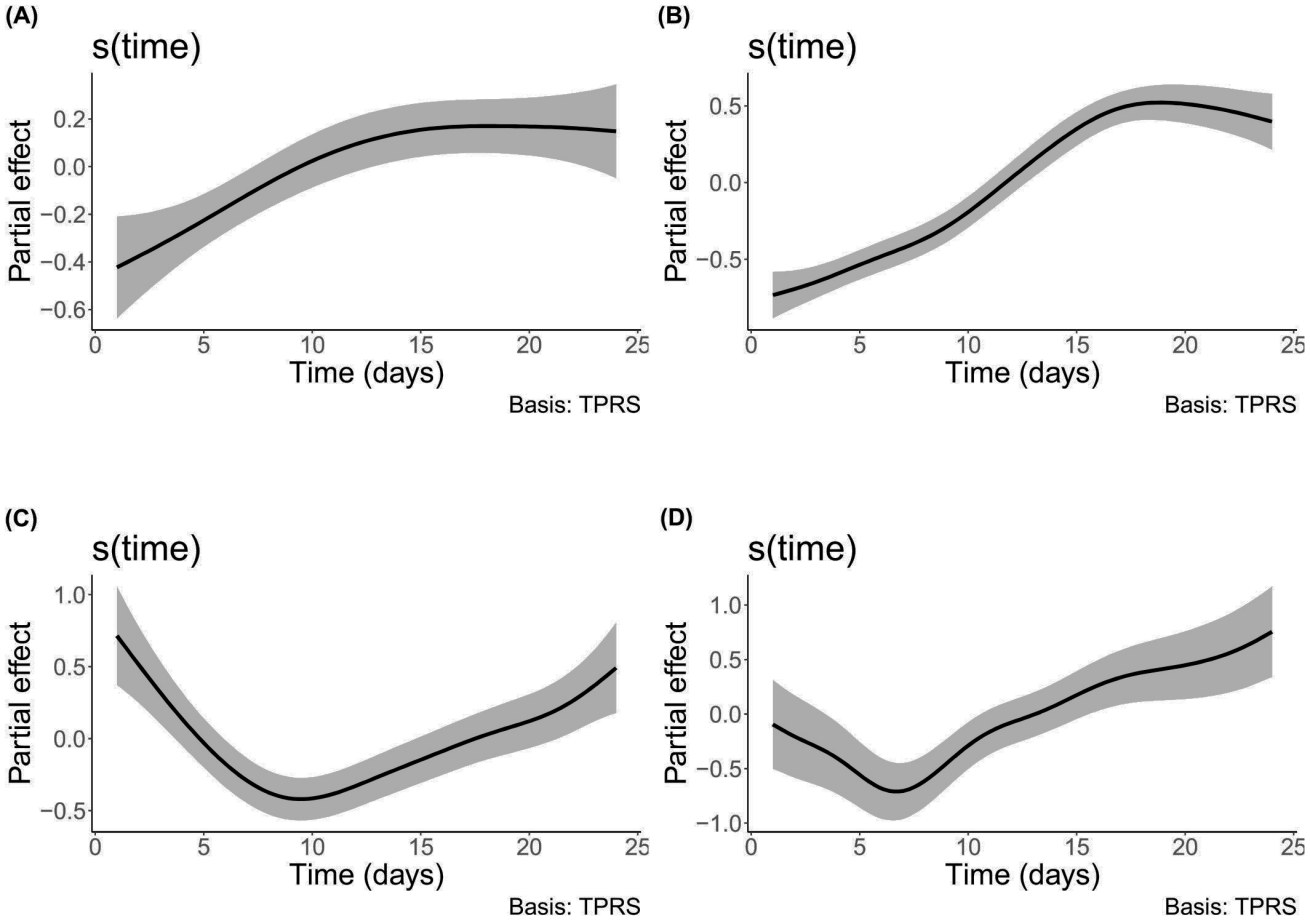
Shared trend of the effect of time on alpha diversity. The shaded area indicates the 0.95 credible interval. **(A)** sessile bacteria of *E1*; **(B)** planktonic bacteria of *E2*; **(C)** sessile eukaryotes of *E1*; and **(D)** planktonic eukaryotes of *E2*.

The shared trend of time was the only parameter of said models that was always significantly correlated to the alpha diversity (Supplementary Table 3). The influence of time on both bacterial sessile and planktonic communities' alpha-diversity indices increased until the 16th day of incubation and stabilized or slightly decreased afterwards (Figures 4A, B). On the other hand, the influence of time on both eukaryotic communities' indices decreased up to day 7 for the planktonic community and day 9 for the sessile community and increased afterwards until the end of the experiment (Figures 4C, D). For both domains, the variations between the minimal and maximal influence of the shared trend of time are greater in the planktonic community than in the sessile one (Supplementary Table 3). Based on this metric, time seems to have been correlated with a higher variation of the alpha diversity in the planktonic community than in the sessile one. The planktonic lifestyle thus may provide a wider ranging availability of niches through time or a varying amount of competition depending on time whereas the sessile community is more stable in the diversity of available niches it provides, supported by the fact that it had a significantly higher evenness (Supplementary Table 4).

It should however be noted that the individual variations of each bioreactor of the planktonic community are more important than in the sessile one. When the unique variations of each bioreactor from the shared trend were significant in the sessile communities, they were following similar trends to the other bioreactors, but with slightly varying amplitudes or added smaller variations whereas one of the three bioreactors diverged in an important way from the two others in both planktonic domains.

The modeled effect of inorganic carbon concentration variation on alpha diversity was significant for the bacterial planktonic community of *E2* where DIC concentration had a rather stable effect until it reached approximately 55 mg/L when the effect then increased until reaching the maximum measured concentration of 73.83 mg/L (Supplementary Figure 8). For the other communities, both organic and inorganic carbon did not have a significant effect on alpha diversity.

### 3.5 NMDS ordination of beta-diversity and sample clustering

**Figure 5:**
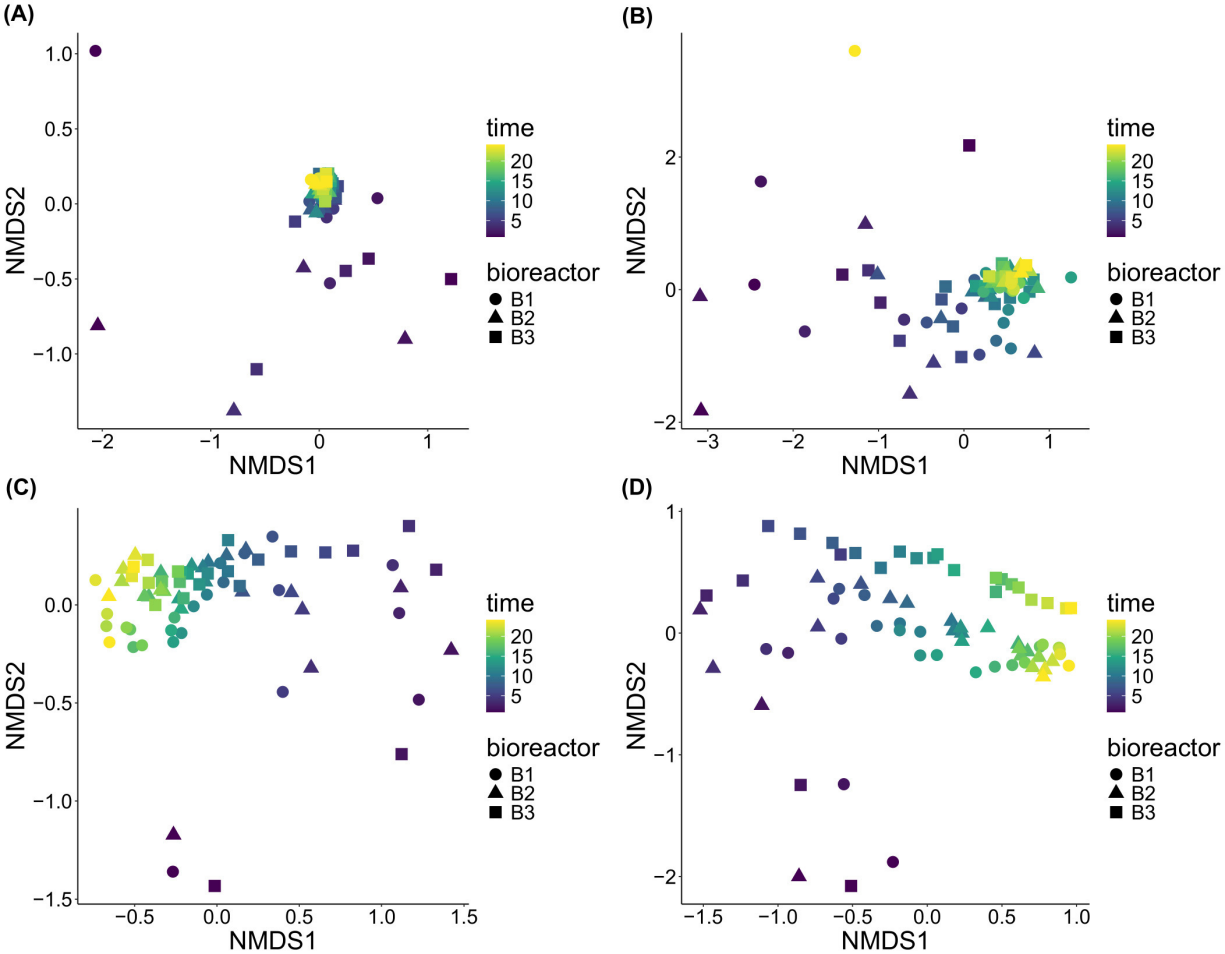
NMDS of the beta diversity of all communities based on Bray-Curtis dissimilarities. **(A)** sessile bacteria of *E1*; **(B)** sessile eukaryotes of *E1*; **(C)** planktonic bacteria of *E2*; and **(D)** planktonic eukaryotes of *E2*.

All the stresses for the sessile and planktonic communities were around 0.1 except for *E1* for the eukaryotes that was of 0.17 (Figure 5). All the communities' variations therefore seem to be reasonably accurately represented by the NMDS graphs. For *E1*, for both domains, the NMDS ordination shows that the early days of incubation led to distinct sessile communities, compared to the later days which clustered closely together (Figures 5A, B). These observations were confirmed by the cluster dendrograms, highlighting a very distinct sessile community for the first and often the 2^nd^ day of incubation for both domains (Supplementary Figure 9). For the bacteria, the dendrograms also show that the second cluster regrouping samples from days 3 or 4 to 24 were subdivided further into 2 clusters and more. However, succession as assessed by these sub-clusters seems to have occurred differently in each bioreactor.

For *E2*, for both domains, succession of planktonic communities appears to be more continuous over time, since the early day samples don't cluster as separately from the later day samples, compared to *E1* (Figures 5C, D). For the Bacteria domain, the cluster dendrograms show indeed one cluster containing the first 4 to 6 days of incubation, and do not show the 1^st^ day standing out as much as for *E1* (Supplementary Figure 9).

With the aim of performing a sensitivity analysis, NMDS analyses using Hellinger transformed data (Supplementary Figure 10) and Bray-Curtis dissimilarity index (Figure 5) were performed. While the Hellinger transformation made the sessile eukaryotes' plot unreadable since most points were stacked on top of each other, it made the similarity between later and earlier samples of both planktonic communities disappear. It therefore seems likely that the similarity between earlier and later samples of the planktonic communities is not ecologically meaningful and solely an artifact of the ordination, namely a horseshoe effect.

### 3.6 Db-RDA, ANOVA, and variance partitioning

All of the samples of both domains were compared using a db-RDA and a one degree of freedom sequential contrast ANOVA (999 permutations) on the db-RDA results. The different lifestyles, experiments and bioreactors were all significantly different (Supplementary Table 5).

The db-RDA followed by the ANOVA and the variance partitioning performed on all the communities indicated that the time was the main driver of variation (Supplementary Table 6). Time explained more of the variation in the planktonic communities than in the sessile ones and it was always a significant explanatory variant in the ANOVA. Based on vector orientation in the db-RDA, time also was positively correlated to varying degrees with organic and inorganic carbon (Supplementary Figure 11). Regarding the individual effect of each bioreactors, they did not influence the community in similar ways (Supplementary Figure 11, Supplementary Table 6).

Regarding the effect of the variation of organic and inorganic carbon concentration on beta diversity, it played a small, but statistically significant role in all communities. The interaction of DIC and DOC concentrations for both domains, however, was not significant (Supplementary Table 6).

### 3.7 Microbial source tracking

After the first few days, where multiple sources were the origins of each community, the community of the same lifestyle of the previous days quickly became, in most cases, the unique source explaining the community of a specific day. There however were days, different for each bioreactor, where another source explained an important part of the community's origin for all communities; days 12 and 22 for the bacterial sessile community of *E1*, days 21 to 23 for the planktonic bacterial community of *E2* and days 5, 9, 12–14 and 18 to 20 for the planktonic eukaryotes. Meanwhile, the sessile eukaryotes did not follow the same pattern. The sessile community of the previous days and the planktonic community alternated to explain a major part of the community and the “unknown” source tended to decrease through time. The planktonic source also generally tends to explain a higher proportion of the community before day eight than after. After this day, despite great variations, the sessile community from the previous days explains a higher proportion of the sessile community. The peat bogs will not be discussed further since they explained 0% of the variation for all communities (Supplementary Figure 12).

## 4 Discussion

Time was shown to be the parameter most correlated with both alpha and beta diversity variations in all communities and the primary succession seems to have been separated into two phases. The first one, where high variation and high diversity were observed, had a duration of approximately 2 days and the second one, where change was much more progressive, lasted for the remainder of the experiment. Sessile bacteria of *E1* underwent succession from chemolithotrophs to heterotrophs and planktonic bacteria mostly underwent a succession of heterotrophs. Regarding eukaryotes, both sessile and planktonic communities saw a succession of likely mixotrophic microorganisms and heterotrophs. The alpha diversity of planktonic and sessile communities of both domains followed similar patterns, but the planktonic communities varied with a broader amplitude. This is opposite to what was expected. Even though they were expected to be weakly active, not multiply that much and originate from the sessile communities, the planktonic communities were correlated with larger variations both in alpha and beta diversity both in Bacteria and Eukaryote domains. Following the first few chaotic days, both domains were well insulated from the sessile communities. The effect of the variation in concentration of DIC and DOC also was much weaker than expected both on alpha and beta diversity. This suggests that in a geochemically stable environment rich in organic carbon and low in nitrogen compounds and electrolytes, planktonic microorganisms are more active than expected in the primary succession and that pH or nutrients other than carbon—such as N, P, S, and Fe restrict the variation of these communities. Further investigation on these communities by following a broader array of nutrients throughout the incubations would help shed light on the unique community dynamics and the influence each nutrient has.

It also highlights the need to use multi-omics approaches to distinguish between sessile and planktonic microorganisms since the current filtering methods used in this study and in most of the literature to analyse planktonic communities do not allow for distinction between planktonic microorganisms and free-floating ones on small sediments.

### 4.1 Temporal primary succession of the sessile community

In *E1*, the sessile communities had a high evenness in the first few days which led to a high alpha diversity index which then decreased as evenness decreased sharply after day 4. The alpha diversity then increased more slowly from day five onwards as richness increased. This may represent the early stages of solid surface colonization (Griebler and Lueders, 2009) where there was less competition between available niches in the beginning of the primary succession in the sessile environment. The early colonizers could therefore multiply at a faster rate. Once the niches began filling up, the communities convened toward a similar beta diversity, like what has been observed in a coal seam (Vick et al., 2019). The evenness decreased until day five and stabilized until the end of the experiment. These changes also coincided with the establishment of a few dominant genera around day 4 and the end of the transition from chemolithototrophs, mainly *Sulfurimonas*, but also *Sideroxydans*, to heterotrophs such as *Pseudomonas*, unc. *Acetobacteraceae*, unc. *Oxalobacteraceae, Undibacterium, Rhodoferax, Polaromonas*, and *Collimonas*. While we could not reliably identify the ASVs to the species level, all these genera contain species capable of forming biofilms (Besemer et al., 2012; Song et al., 2015; Thi et al., 2020). Thus, biofilm formation may have occurred as soon as the cell density was sufficient.

Regarding the sessile eukaryotes of *E1*, the first few days (2 to 4 depending on the bioreactor) of the succession in the communities saw rapid taxonomical changes, going from being comprised of fungi, protists and many other heterotrophs to being mostly composed of the potentially photosynthetic autotrophs unc. *Synuracea* and unc. *Chrysophyceae*. It should however be noted that some of these microorganisms may have mixotrophic capabilities (Cavalier-Smith and Chao, 2006; Siver, 2003). *Sandonidae*_X also increased in relative abundance followed by the bacterivorous *Apoikiospumella*. Algae always occupied a predominant place in the community, occupying above 50% of the relative abundance for most of the days. This predominance of potentially photosynthetic or mixotrophic microorganisms is rather surprising since there was a high concentration of available carbon to be consumed by heterotrophs and the incubations took place in a dark growth chamber, the bioreactors used for the incubation were wrapped in aluminum foil and the bottles used to feed water into the system were also obscured. The tubes connecting the bioreactors to the water collection bottles and the collection bottles themselves were, however, not obscured and may have let some light into the system whenever the growth chamber's doors were opened daily for sampling. However, the fact that we did not observe similar metabolic taxa in the Bacteria domain despite the presence of cyanobacteria, *Rhodoblastus* and other photosynthetic genera in the initial *in situ* groundwater would make the mixotrophic explanation more likely.

After ca. 6 days, the bacterial sessile communities originated mostly from the sessile communities of the previous days. The critical days determining the future characteristics of a community would therefore be the first few days with the community of the subsequent days being fairly well insulated from the other ones and the internal dynamics of the community determining its changes. The groundwater during the incubations and the groundwater collected on the sampling day seem to exceptionally have had an important influence on days 12 and 22. As long as the geochemical environment is kept stable, the community thus seems to be fairly well insulated from outside influence except on a few specific days. The sessile eukaryotes of *E1* are the exception to this insulation. They did not show any particular patterns in all three bioreactors, alternating between being mostly explained by the sessile community of the previous days and the planktonic community. The decreasing influence of the planktonic community after day 8 may however indicate that this community, if given enough time, would stabilize itself as the others did. It also should be noted that the interactions between the heterotrophic and mixotrophic eukaryotes and the various prokaryotes and dissolved organic carbon species available as carbon sources most likely played a role in shaping the sessile communities (Herrmann et al., 2020) and should be investigated further. Especially given the presence of fungal taxa competing for dissolved organic carbon with heterotrophic bacteria and of *Apoikiospumella*, a bacterivorous taxon (Grossmann et al., 2016).

### 4.2 Temporal primary succession of the planktonic community

In the planktonic bacterial community of *E2*, the alpha diversity variation through time followed similar patterns as in the sessile bacterial community of *E1* albeit with a broader amplitude. There also doesn't seem to have been a first period of the succession where autotrophic microorganisms played an important role in the community, there only was a succession of heterotrophs in the dominant genera. There thus seems to have been a different response between the sessile and planktonic communities regarding metabolic diversity during the community succession. Also, as previously observed, the observed richness of the planktonic communities was much higher than the one of the sessile communities (Patel et al., 2024; Sharma et al., 2024; Yan et al., 2020), especially in the first few days. This high richness led to a higher Shannon index and, following the strong decrease in richness during the 1^st^ or 2^nd^ day, the evenness increased, but more slowly. This also led to an increase in the Shannon index. The planktonic communities thus were composed of a wide range of genera with highly varying abundances in the early days, but the number of genera quickly decreased, and the fewer remaining genera had a progressively more equal distribution as the experiment progressed.

Regarding the planktonic eukaryotes of *E2*, likely mixotrophic microorganisms also dominated most of the incubation despite the presence of heterotrophs and of high organic carbon concentrations. *Spumella, Vorticella* and unc. *Synuraceae* represented the vast majority of the community for most of the experiment and *Apoikiospumella* increased strongly in abundance in the latter half of the experiment. This highlights once again the importance of better understanding the interaction dynamics between heterotrophs and mixotrophs to better understand these environments.

The planktonic eukaryotes of *E2* displayed a behavior similar to both bacterial communities where, following the first few chaotic days, the planktonic community of the previous days explained almost all of the community of a given day while the community of the sessile eukaryotes of *E1* were frequently influenced by the planktonic community colonizing them. It thus seems that planktonic eukaryotes would frequently but briefly interact with the sessile communities in *E1*, but the sessile microorganisms would seldom go into the planktonic community in *E2*. We however only have two time point for the sessile and planktonic interactions in *E2* so further investigation is warranted.

The planktonic communities of *E1* were extracted directly from the aquifer and thus had an unknown age whereas the planktonic communities of *E2* were undergoing primary succession starting on the 1^st^ day of the second phase. It therefore is not surprising for both of them to have had a markedly different behavior throughout the 24 days of the incubation. In addition to being taxonomically distinct, the planktonic communities extracted from the aquifer used to colonize the rock pellets of *E1* displayed a more stable behavior with less fluctuation in genera than in the planktonic communities of *E2*. These older communities had likely reached an equilibrium that could not be reached within 24 days of the beginning of primary succession for the planktonic community of *E2* (Fillinger et al., 2018).

### 4.3 Sessile and planktonic communities: how do they differ and how are they related?

Since the sessile communities were expected to be more active than the planktonic ones and increase in abundance faster, it was expected for time to play a more important role in the sessile communities than in the planktonic ones. However, we observed the opposite trend: while time correlated in a small but significant way with the beta diversity of all communities, it explained a bigger part of the variation of the planktonic communities than of the sessile ones. The planktonic communities also, as expected, had a higher richness (Patel et al., 2024; Sharma et al., 2024; Yan et al., 2020), and a higher share of unique ASVs (Sharma et al., 2024). This may indicate that the planktonic lifestyle provided a wide-ranging availability of niches depending on time whereas the higher evenness of the sessile community indicates a higher stability in the provided niches.

The high proportion of ASVs present in the colonized community, but not in the colonizing community also highlights the challenges associated with detecting scarce ASVs. For example, the ASVs detected in the planktonic communities of *E2* most likely fell below the detection threshold in the colonizing sessile communities but increased in abundance once in the planktonic communities. It also is possible, albeit less likely, for the 16S rRNA genes to have been involved in horizontal gene transfer (Bartoš et al., 2024) which would similarly explain the presence of so many unique ASVs to each community. The protocol used to extract DNA from the planktonic community may also have been more effective or extracted fewer mineral impurities which would likely have influenced the effectiveness of the PCR and the sequencing.

Regarding the source of each community, after the first few highly variable days, all communities except the eukaryotic sessile communities' source could mostly be attributed to their community from the previous day. This suggests that most communities in stable biogeochemical conditions were well insulated from outside biotic influence, but repeated experiments are needed to determine if the eukaryotic sessile communities are repeatedly much less constant in the origin of their communities or if the observed trend was an anomaly.

The fact that similar behavior has been observed in all three bioreactors, however, seems to indicate that this is to be expected. It may have been caused by eukaryotes interacting with a bacterial biofilm but being unable to form one themselves (Zirnstein et al., 2012) and thus, being more or less well insulated from the planktonic community. That would explain why the planktonic community was the source of the sessile community in the early days and, following the establishment of the biofilm, the microorganisms that positively interacted with the biofilm were generally better insulated from outside influence following day 8 despite important planktonic influences still occurring on some days. This general trend of communities changing through time and being explained almost entirely by their own communities of the previous days also comes in stark contradiction to the literature that expects the planktonic community to be populated by weakly active microorganisms (Griebler and Lueders, 2009; Sharma et al., 2024; Wilhartitz et al., 2009) that, in the context of primary succession, would come from the sessile community.

As observed for the alpha diversity of the planktonic communities where time was correlated with a stronger variation of alpha diversity, the beta diversity of the planktonic communities had a stronger correlation with time than the sessile ones. This is opposite to our initial hypothesis as it was expected that the sessile community would be more active and faster growing and changing. Such expectations were, however, based on studies led in a different, limestone-rich environments (Sharma et al., 2024; Wilhartitz et al., 2009). This difference in the geological environment may explain why the microorganism's behavior differed from what we observed. The general trend nonetheless was for a stronger variation of planktonic communities.

### 4.4 Influence of environmental parameters on microbial communities

Since *in situ* and incubation conditions differed between both sampling times, it unsurprisingly led to different communities. All communities were nonetheless incubated in an oxic environment rich in dissolved organic and inorganic carbon, but poor in nitrogen compounds and their temporal dynamics and taxonomical composition were similar.

The DIC and DOC concentrations were high, and their variations correlated with the alpha and beta diversity variations in a much weaker way than expected. Both organic and inorganic carbon concentration variations explained a higher proportion of the variation of the beta diversity of the eukaryotes than of the bacteria. This is opposite to the influence of inorganic carbon concentration variation on alpha diversity that explained a bigger proportion of the bacteria than the eukaryotes. This may therefore mean that the variation in DIC concentration correlated to stark changes in the composition of the community (alpha diversity) of the planktonic bacterial community, but these changes were weaker between the different days sampled (beta diversity).

It also was expected for the variation in concentration of organic carbon to have an influence on the composition of the communities and especially on the shift from autotrophs to heterotrophs. While its high concentration most likely caused heterotrophs to quickly dominate the chemolithotrophs (Fierer et al., 2010), its variation does not seem to have had an important influence. This may be because, with a minimum DOC concentration for the first experiment of 24 mg/L and of 16 mg/L for the second, these concentrations were much higher than the rough approximation of maximum 4 mg/L of DOC that is usually found in groundwater (Regan et al., 2017). While there is no known contamination source for the aquifer we sampled (Chabot-Grégoire, 2024), these much higher than normal concentrations usually are indicative of contamination (Regan et al., 2017). The nitrogen levels measured in the aquifer, however, were low, as usually observed in pristine aquifers (Kumar et al., 2017; Morgenstern and Daughney, 2012) and may have restricted community growth (Schwab et al., 2016). The analysis of nitrogen concentration throughout the incubation would allow us to observe more precisely its variations in concentration and its subsequent influence on the community as it likely was an important factor restricting community growth. The low conductivity levels measured *in situ* also indicate low concentration of electrolytes in the water (Gray, 2004) so other electrolytes such as Fe (Jakus et al., 2021), P (Rogers et al., 1998), and S (Labrenz et al., 2013) compounds may have been in low concentration and restricting growth.

Regarding the planktonic bacterial community, the correlation between its variation and the variation in concentration of inorganic carbon may be explained by the fact that, in an acidic environment such as the groundwater we used for our experiment, most of the inorganic carbon present in solution is carbonic acid (dissolved CO_2_) (Cole and Prairie, 2024). Since the heterotrophs composing most of the community do not use inorganic carbon as a carbon source, this correlation between DIC concentration variation and the planktonic bacterial community of *E2* may be due to the effect the variations in CO_2_ concentration had on the variation in pH and subsequently on the growing conditions of the microorganisms of the community. The measured pH of the second experiment (pH: min = 4.17, median = 4.41, max = 4.89) indeed remained much below the optimal pH of the most abundant prokaryotic taxa (Belmok et al., 2023; Hommel and Ahnert, 1999). While the optima cited in the literature generally are for specific species and the microorganisms could not reliably be identified to the species level, it supports the possibility that the fluctuations in pH measured in the community generally may have limited and influenced the growth of the communities whereas the fluctuations of DOC concentration did not since DOC's absolute concentration always was fairly high. It also would explain why only the higher concentrations of DIC affected the alpha diversity of the community; the pH had to be below a certain threshold to influence in an important way the microorganisms during the incubation. Similarly, the eukaryotic dominant genera may have been limited by pH (Moser and Weisse, 2011; Sudo and Aiba, 1973), low nitrogen concentrations and the concentration of other nutrients (Poikane et al., 2022). The overbearing influence of these factors would explain the weak correlations between carbon concentration variation and diversity as these other parameters determined the changes occurring in the community. The proportion to which mixotrophy was an important strategy in the community also has yet to be established and likely played a crucial role in shaping it.

### 4.5 Methodological limitations

All of these distinctions noted between sessile and planktonic communities and contradictions between them and the literature may be explained by the fact that we kept the geochemical parameters as stable as possible throughout the experiment, studied both prokaryotes and eukaryotes and studied the short term primary succession while studies generally analyse communities over longer periods and in changing environments (Dong et al., 2021; Fillinger et al., 2021, 2018). The trends observed in these studies may therefore not directly translate to ours since the geochemical variations tends to exert a strong influence on these communities. The studies with closer methodology to ours are extremely rare (Patel et al., 2024; Sharma et al., 2024) and took a more limited interest into the planktonic and eukaryotic communities and so, comparison is more difficult.

It also should be noted that we classified the planktonic communities as the ones extracted from the filters and the sessile communities as the ones that were attached to the rock pellets, as is usually the case (Fillinger et al., 2021; Gios et al., 2023; Patel et al., 2024; Sharma et al., 2024; Yan et al., 2020). This means that we could not differentiate between the free-floating microorganisms and the ones living a sessile lifestyle on small free-floating sediments. It therefore cannot be ruled out that the sessile microorganisms living on flowing sediments were the most active sub-community that was studied from the groundwater and the planktonic community *per se* only was weakly active, as has been observed in the literature. There also likely was a community of ultra-small bacteria that could not be studied since they were smaller than the 0.2 μm pores of our filters and thus, were not detected by our methods (Herrmann et al., 2019). Multi-omics methods enable to better understand the distinction between these varying communities (Atencio et al., 2025; Smith et al., 2018), but, given the challenging DNA extraction and purification of the microorganisms living in these environments, the frequent co-extraction of PCR inhibitors and the small knowledge we have of these unique taxa, such methods are seldom used.

## 5 Conclusions

To summarize, time was consistently correlated to the variations in the diversity of the communities. Its influence varied based on the lifestyle and the domain, but it always was the parameter with the strongest correlation with diversity variation. The alpha diversity of the planktonic communities varied more through time than the sessile ones. The variation in the studied concentrations of inorganic carbon displayed a much weaker correlation than expected with alpha and beta diversity and was mostly relevant to explain the variation in the alpha diversity of the prokaryotic planktonic community. When analyzed, the variation in organic carbon concentration failed to show an important influence on the communities, but its high concentration likely played an important role in determining the composition and temporal dynamics of the community. This much weaker than expected influence of carbon concentration variation is likely due to the low pH of the environment or the concentration of other nutrients such as nitrogen restricting the development of these communities.

This study is the first we could find that analyzed the daily succession and interactions of subsurface bacteria and eukaryotes in sessile and planktonic communities. It enabled a higher temporal resolution of the subsurface communities' dynamics and reduced the influence of variation in concentration of geo-chemical parameter. The high concentration of organic carbon and low concentration of nitrogen, however, likely exerted a strong influence on the composition of the communities and their dynamics. We were able to observe the clear demarcation, especially in the bacteria, of the primary succession into two distinct temporal phases. The first was of a few days where high variability prevailed. The second phase, of which we could not observe the end, was characterized by slower changes. Regarding community composition, the first phase contained bacterial chemolithotrophs whereas the second phase was almost exclusively bacterial heterotrophs. The eukaryotes did not show such a stark contrast in the variation of community composition between the two phases, displaying likely mixotrophic taxa throughout the incubation. These communities' temporal dynamics and the interactions between prokaryotes and eukaryotes still are little understood and warrant further investigation in varying nutrient concentrations and, if possible, using multi-omics, to better understand how different perturbations may affect these subsurface communities and how they may subsequently respond.

## Data Availability

The datasets generated for and analyzed in this study can be found in the National Center for Biotechnology Information (NCBI) under the BioProject ID (PRJNA1159903).

## References

[B1] Atencio B. Malavin S. Rubin-Blum M. Ram R. Adar E. Ronen Z. (2025). Site-specific incubations reveal biofilm diversity and functional adaptations in deep, ancient desert aquifers. Front. Microbiol. 16:1533115. 10.3389/fmicb.2025.1533115 40190731 PMC11968702

[B2] Bartoš O. Chmel M. Swierczková I. (2024). The overlooked evolutionary dynamics of 16S rRNA revises its role as the “gold standard” for bacterial species identification. Sci. Rep. 14:9067. 10.1038/s41598-024-59667-3 38643216 PMC11032355

[B3] Belmok A. de Almeida F. M. Rocha R. T. Vizzotto C. S. Tótola M. R. Ramada M. H. S.. (2023). Genomic and physiological characterization of *Novosphingobium terrae* sp. nov., an alphaproteobacterium isolated from Cerrado soil containing a mega-sized chromid. Brazil. J. Microbiol. 54, 239–258. 10.1007/s42770-022-00900-4 36701110 PMC9944591

[B4] Besemer K. Peter H. Logue J. B. Langenheder S. Lindström E. S. Tranvik L. J.. (2012). Unraveling assembly of stream biofilm communities. ISME J. 6, 1459–1468. 10.1038/ismej.2011.205 22237539 PMC3400417

[B5] Callahan B. J. McMurdie P. J. Rosen M. J. Han A. W. Johnson A. J. A. Holmes S. P. (2016). DADA2: High-resolution sample inference from Illumina amplicon data. Nat. Methods 13, 581–583. 10.1038/nmeth.3869 27214047 PMC4927377

[B6] Cavalier-Smith T. Chao E. E.-Y. (2006). Phylogeny and megasystematics of phagotrophic heterokonts (Kingdom Chromista). J. Mol. Evol. 62, 388–420. 10.1007/s00239-004-0353-8 16557340

[B7] Chabot-Grégoire J. (2024). Email to Samuel Beauregard-Tousignant, 13 March.

[B8] Cole J. J. Prairie Y. T. (2024). “The inorganic carbon complex,” *Wetzel's Limnology*, 301–323. 10.1016/B978-0-12-822701-5.00013-6

[B9] Davis N. M. Proctor D. M. Holmes S. P. Relman D. A. Callahan B. J. (2018). Simple statistical identification and removal of contaminant sequences in marker-gene and metagenomics data. Microbiome 6:226. 10.1186/s40168-018-0605-2 30558668 PMC6298009

[B10] del Campo J. Kolisko M. Boscaro V. Santoferrara L. F. Nenarokov S. Massana R.. (2018). EukRef: Phylogenetic curation of ribosomal RNA to enhance understanding of eukaryotic diversity and distribution. PLoS Biol. 16:e2005849. 10.1371/journal.pbio.2005849 30222734 PMC6160240

[B11] Dixon P. (2003). VEGAN, a package of R functions for community ecology. J. Veget. Sci. 14, 927–930. 10.1111/j.1654-1103.2003.tb02228.x

[B12] Dong Y. Sanford R. A. Connor L. Chee-Sanford J. Wimmer B. T. Iranmanesh A.. (2021). Differential structure and functional gene response to geochemistry associated with the suspended and attached shallow aquifer microbiomes from the Illinois Basin, IL. Water Res. 202:117431. 10.1016/j.watres.2021.117431 34320445

[B13] Emerson J. B. Thomas B. C. Alvarez W. Banfield J. F. (2016). Metagenomic analysis of a high carbon dioxide subsurface microbial community populated by chemolithoautotrophs and bacteria and archaea from candidate phyla. Environ. Microbiol. 18, 1686–1703. 10.1111/1462-2920.12817 25727367

[B14] Fierer N. Nemergut D. Knight R. Craine J. M. (2010). Changes through time: integrating microorganisms into the study of succession. Res. Microbiol. 161, 635–642. 10.1016/j.resmic.2010.06.002 20599610

[B15] Fillinger L. Hug K. Griebler C. (2021). Aquifer recharge viewed through the lens of microbial community ecology: Initial disturbance response, and impacts of species sorting versus mass effects on microbial community assembly in groundwater during riverbank filtration. Water Res. 189:116631. 10.1016/j.watres.2020.116631 33217664

[B16] Fillinger L. Zhou Y. Kellermann C. Griebler C. (2018). Non-random processes determine the colonization of groundwater sediments by microbial communities in a pristine porous aquifer. Environ. Microbiol. 21, 327–342. 10.1111/1462-2920.14463 30378251

[B17] Flynn T. M. Sanford R. A. Ryu H. Bethke C. M. Levine A. D. Ashbolt N. J.. (2013). Functional microbial diversity explains groundwater chemistry in a pristine aquifer. BMC Microbiol. 13:146. 10.1186/1471-2180-13-146 23800252 PMC3700874

[B18] Gios E. Mosley O. E. Weaver L. Close M. Daughney C. Handley K. M. (2023). Ultra-small bacteria and archaea exhibit genetic flexibility towards groundwater oxygen content, and adaptations for attached or planktonic lifestyles. ISME Commun. 3:13. 10.1038/s43705-023-00223-x 36808147 PMC9938205

[B19] Girard P. Levison J. Parrott L. Larocque M. Ouellet M.-A. Green D. M. (2015). Modeling cross-scale relationships between climate, hydrology, and individual animals: generating scenarios for stream salamanders. Front. Environ. Sci. 3:51. 10.3389/fenvs.2015.00051

[B20] Glöckner F. O. Yilmaz P. Quast C. Gerken J. Beccati A. Ciuprina A.. (2017). 25 years of serving the community with ribosomal RNA gene reference databases and tools. J. Biotechnol. 261, 169–176. 10.1016/j.jbiotec.2017.06.1198 28648396

[B21] Gray J. R. (2004). “Conductivity analyzers and their application,” *Environmental Instrumentation and Analysis Handbook*, 491–510. 10.1002/0471473332.ch23

[B22] Griebler C. Lueders T. (2009). Microbial biodiversity in groundwater ecosystems. Freshw. Biol. 54, 649–677. 10.1111/j.1365-2427.2008.02013.x

[B23] Grossmann L. Bock C. Schweikert M. Boenigk J. (2016). Small but Manifold – Hidden Diversity in “Spumella-like Flagellates.” J. Eukaryotic Microbiol. 63, 419–439. 10.1111/jeu.12287 26662881 PMC5066751

[B24] Guillou L. Bachar D. Audic S. Bass D. Berney C. Bittner L.. (2012). The Protist Ribosomal Reference database (PR2): a catalog of unicellular eukaryote Small Sub-Unit rRNA sequences with curated taxonomy. Nucleic Acids Res. 41, D597–D604. 10.1093/nar/gks1160 23193267 PMC3531120

[B25] Herrmann M. Geesink P. Yan L. Lehmann R. Totsche K. U. Küsel K. (2020). Complex food webs coincide with high genetic potential for chemolithoautotrophy in fractured bedrock groundwater. Water Res. 170:115306. 10.1016/j.watres.2019.115306 31770650

[B26] Herrmann M. Opitz S. Harzer R. Totsche K. Küsel K. (2017). Attached and suspended denitrifier communities in pristine limestone aquifers harbor high fractions of potential autotrophs oxidizing reduced iron and sulfur compounds. Microb. Ecol. 74, 264–277. 10.1007/s00248-017-0950-x 28214969

[B27] Herrmann M. Wegner C.-E. Taubert M. Geesink P. Lehmann K. Yan L.. (2019). Predominance of cand. patescibacteria in groundwater is caused by their preferential mobilization from soils and flourishing under oligotrophic conditions. Front. Microbiol. 10:1407. 10.3389/fmicb.2019.01407 31281301 PMC6596338

[B28] Hommel R. K. Ahnert P. (1999). “Acetobacter,” in Encyclopedia of Food Microbiology, ed. R. K. Robinson (Oxford: Elsevier). 10.1006/rwfm.1999.0005

[B29] Hoshino T. Inagaki F. (2018). Abundance and distribution of Archaea in the subseafloor sedimentary biosphere. ISME J. 13, 227–231. 10.1038/s41396-018-0253-3 30116037 PMC6298964

[B30] Hull N. M. Rosenblum J. S. Robertson C. E. Harris J. K. Linden K. G. (2018). Succession of toxicity and microbiota in hydraulic fracturing flowback and produced water in the Denver–Julesburg Basin. Sci. Total Environ. 644, 183–192. 10.1016/j.scitotenv.2018.06.067 29981518

[B31] Jakus N. Blackwell N. Osenbrück K. Straub D. Byrne J. M. Wang Z.. (2021). Nitrate removal by a novel lithoautotrophic nitrate-reducing, Iron(II)-oxidizing culture enriched from a pyrite-rich limestone aquifer. Appl. Environ. Microbiol. 87, e00460–e00421. 10.1128/AEM.00460-21 34085863 PMC8373248

[B32] Jewell T. N. M. Karaoz U. Brodie E. L. Williams K. H. Beller H. R. (2016). Metatranscriptomic evidence of pervasive and diverse chemolithoautotrophy relevant to C, S, N and Fe cycling in a shallow alluvial aquifer. ISME J. 10, 2106–2117. 10.1038/ismej.2016.25 26943628 PMC4989316

[B33] Jin C. Sengupta A. (2024). Microbes in porous environments: from active interactions to emergent feedback. Biophys. Rev. 16, 173–188. 10.1007/s12551-024-01185-7 38737203 PMC11078916

[B34] Jowers M. J. Xavier R. Lasso-Alcal,á O. M. Quintero-T E Nunes J. L. S.. (2023). First molecular identification of a Goussia parasite from a new world invasive blenny. Acta Parasitol. 68, 458–462. 10.1007/s11686-023-00675-0 37103766

[B35] Katayama T. Ikawa R. Koshigai M. Sakata S. (2023). Microbial methane formation in deep aquifers associated with the sediment burial history at a coastal site. Biogeosciences 20, 5199–5210. 10.5194/bg-20-5199-2023

[B36] Kumar S. Herrmann M. Thamdrup B. Schwab V. F. Geesink P. Trumbore S. E.. (2017). Nitrogen loss from pristine carbonate-rock aquifers of the hainich critical zone exploratory (Germany) is primarily driven by chemolithoautotrophic anammox processes. Front. Microbiol. 8:1951. 10.3389/fmicb.2017.01951 29067012 PMC5641322

[B37] Labrenz M. Grote J. Mammitzsch K. Boschker H. T. S. Laue M. Jost G.. (2013). *Sulfurimonas gotlandica* sp. nov., a chemoautotrophic and psychrotolerant epsilonproteobacterium isolated from a pelagic redoxcline, and an emended description of the genus Sulfurimonas. Int. J. System. Evolut. Microbiol. 63, 4141–4148. 10.1099/ijs.0.048827-0 23749282 PMC3836495

[B38] Lee M. D. Pedroso A. A. Maurer J. J. (2023). Bacterial composition of a competitive exclusion product and its correlation with product efficacy at reducing Salmonella in poultry. Front. Physiol. 13:1043383. 10.3389/fphys.2022.1043383 36699689 PMC9868637

[B39] Levison J. Larocque M. Fournier V. Gagné S. Pellerin S. Ouellet M. A. (2013). Dynamics of a headwater system and peatland under current conditions and with climate change. Hydrol. Process. 28, 4808–4822. 10.1002/hyp.9978

[B40] Marshall K. C. (2013). “Planktonic versus sessile life of prokaryotes,” in The Prokaryotes: Prokaryotic Communities and Ecophysiology, eds. E. Rosenberg, E. F. Delong, S. Lory, E. Stackebrandt and F. Thompson (Berlin, Heidelberg: Springer Berlin Heidelberg).

[B41] McMurdie P. J. Holmes S. (2013). phyloseq: an R package for reproducible interactive analysis and graphics of microbiome census data. PLoS ONE 8:e61217. 10.1371/journal.pone.0061217 23630581 PMC3632530

[B42] Morgenstern U. Daughney C. J. (2012). Groundwater age for identification of baseline groundwater quality and impacts of land-use intensification – The National Groundwater Monitoring Programme of New Zealand. J. Hydrol. 456, 79–93. 10.1016/j.jhydrol.2012.06.010

[B43] Moser M. Weisse T. (2011). The outcome of competition between the two chrysomonads *Ochromonas* sp. and *Poterioochromonas malhamensis* depends on pH. Eur. J. Protistol. 47, 79–85. 10.1016/j.ejop.2011.01.001 21334865 PMC3117142

[B44] Nastev M. Morin R. Godin R. Rouleau A. (2008). Developing conceptual hydrogeological model for Potsdam sandstones in southwestern Quebec, Canada. Hydrogeol. J. 16, 373–388. 10.1007/s10040-007-0267-9

[B45] Opitz S. Küsel K. Spott O. Totsche K. U. Herrmann M. (2014). Oxygen availability and distance to surface environments determine community composition and abundance of ammonia-oxidizing prokaroytes in two superimposed pristine limestone aquifers in the Hainich region, Germany. FEMS Microbiol. Ecol. 90, 39–53. 10.1111/1574-6941.12370 24953994

[B46] Patel D. Blouin V. Kirkpatrick J. Lazar C. S. (2024). Rock surface colonization by groundwater microorganisms in an aquifer system in Quebec, Canada. Diversity 16:374. 10.3390/d16070374

[B47] Patton C. J. Kryskalla J. R. (2003). “Methods of analysis by the U.S. Geological Survey National Water quality laboratory: evaluation of alkaline per sulfate digestion as an alternative to Kjeldahl digestion for determination of total and dissolved nitrogen and phosphorus in water,” in Water-Resources Investigations Report 2003-4174. USGS Publications Warehouse. 10.3133/WRI034174

[B48] Pearce J. K. Hofmann H. Baublys K. Golding S. D. Rodger I. Hayes P. (2023). Sources and concentrations of methane, ethane, and CO2 in deep aquifers of the Surat Basin, Great Artesian Basin. Int. J. Coal Geol. 265:104162. 10.1016/j.coal.2022.104162

[B49] Pedersen E. J. Miller D. L. Simpson G. L. Ross N. (2019). Hierarchical generalized additive models in ecology: an introduction with mgcv. PeerJ. 7:e6876. 10.7717/peerj.6876 31179172 PMC6542350

[B50] Poikane S. Kelly M. G. Várbíró G. Borics G. Eros T. Hellsten S.. (2022). Estimating nutrient thresholds for eutrophication management: novel insights from understudied lake types. Sci. Total Environ. 827:154242. 10.1016/j.scitotenv.2022.154242 35245557

[B51] Quast C. Pruesse E. Yilmaz P. Gerken J. Schweer T. Yarza P.. (2012). The SILVA ribosomal RNA gene database project: improved data processing and web-based tools. Nucleic Acids Res. 41, D590–D596. 10.1093/nar/gks1219 23193283 PMC3531112

[B52] R Core Team (2024). R: A Language and Environment for Statistical Computing. Vienna, Austria: R Foundation for Statistical Computing. Available online at: https://www.R-project.org/ (accessed March 1, 2025).

[B53] Rajala P. Bomberg M. (2023). Editorial: Geomicrobes: Life in terrestrial deep subsurface, volume II. Front. Microbiol. 14:1169127. 10.3389/fmicb.2023.1169127 36998391 PMC10042722

[B54] Regan S. Hynds P. Flynn R. (2017). Um panorama sobre carbono dissolvido em águas subterrâneas e implicações para segurança da água potável. Hydrogeol. J. 25, 959–967. 10.1007/s10040-017-1583-3

[B55] Rogers J. R. Bennett P. C. Choi W. J. (1998). Feldspars as a source of nutrients for microorganisms. Am. Mineral. 83, 1532–1540. 10.2138/am-1998-11-1241

[B56] Schwab V. F. Hermann M. Roth V.-N. Gleixner G. Lehmann R. Pohnert G.. (2016). Functional diversity of microbial communities in pristine aquifers inferred by PLFA – and sequencing – based approaches. Biogeosciences 14, 2697–2714. 10.5194/bg-14-2697-2017

[B57] Schwab V. F. Nowak M. E. Elder C. D. Trumbore S. E. Xu X. Gleixner G.. (2019). 14C-free carbon is a major contributor to cellular biomass in geochemically distinct groundwater of shallow sedimentary bedrock aquifers. Water Resour. Res. 55, 2104–2121. 10.1029/2017WR022067 31068736 PMC6487957

[B58] Sharma A. Taubert M. Pérez-Carrascal O. M. Lehmann R. Ritschel T. Totsche K. U.. (2024). Iron coatings on carbonate rocks shape the attached bacterial aquifer community. Sci. Total Environ. 917:170384. 10.1016/j.scitotenv.2024.170384 38281639

[B59] Shenhav L. Thompson M. Joseph T. A. Briscoe L. Furman O. Bogumil D.. (2019). FEAST: fast expectation-maximization for microbial source tracking. Nat. Methods 16, 627–632. 10.1038/s41592-019-0431-x 31182859 PMC8535041

[B60] Siver P. A. (2003). “Synurophyte Algae,” in Freshwater Algae of North America, 523–557. 10.1016/B978-012741550-5/50015-5

[B61] Smith H. J. Zelaya A. J. De León K. B. Chakraborty R. Elias D. A. Hazen T. C.. (2018). Impact of hydrologic boundaries on microbial planktonic and biofilm communities in shallow terrestrial subsurface environments. FEMS Microbiol. Ecol. 94:fiy191. 10.1093/femsec/fiy191 30265315 PMC6192502

[B62] Song C. Schmidt R. de Jager V. Krzyzanowska D. Jongedijk E. Cankar K.. (2015). Exploring the genomic traits of fungus-feeding bacterial genus Collimonas. BMC Genomics 16:1103. 10.1186/s12864-015-2289-3 26704531 PMC4690342

[B63] Spring S. Riedel T. Spröer C. Yan S. Harder J. Fuchs B. M. (2013). Taxonomy and evolution of bacteriochlorophyll a-containing members of the OM60/NOR5 clade of marine gammaproteobacteria: description of Luminiphilus syltensis gen. nov., sp. nov., reclassification of *Haliea rubra* as *Pseudohaliea rubra* gen. nov., comb. nov., and emendation of Chromatocurvus halotolerans. BMC Microbiol. 13:118. 10.1186/1471-2180-13-118 23705883 PMC3679898

[B64] Sudo R. Aiba S. (1973). Mass and monoxenic culture of vorticella microstoma isolated from activated sludge. Water Res. 7, 615–621. 10.1016/0043-1354(73)90061-4

[B65] Thi M. T. T. Wibowo D. Rehm B. H. A. (2020). Pseudomonas aeruginosa Biofilms. Int. J. Mol. Sci. 21:8671. 10.3390/ijms21228671 33212950 PMC7698413

[B66] Vaulot D. Geisen S. Mahé F. Bass D. (2021). pr2-primers: An 18S rRNA primer database for protists. Mol. Ecol. Resour. 22, 168–179. 10.1111/1755-0998.13465 34251760

[B67] Vaulot D. Sim C. W. H. Ong D. Teo B. Biwer C. Jamy M.. (2022). metaPR2: a database of eukaryotic 18S rRNA metabarcodes with an emphasis on protists. Mol. Ecol. Resour. 22, 3188–3201. 10.1111/1755-0998.13674 35762265 PMC9796713

[B68] Vick S. H. W. Greenfield P. Pinetown K. L. Sherwood N. Gong S. Tetu S. G.. (2019). Succession patterns and physical niche partitioning in microbial communities from subsurface coal seams. IScience 12, 152–167. 10.1016/j.isci.2019.01.011 30685711 PMC6354743

[B69] Visser A.-N. Martin J. D. Osenbrück K. Rügner H. Grathwohl P. Kappler A. (2024). In situ incubation of iron(II)-bearing minerals and Fe(0) reveals insights into metabolic flexibility of chemolithotrophic bacteria in a nitrate polluted karst aquifer. Sci. Total Environ. 926:172062. 10.1016/j.scitotenv.2024.172062 38554974

[B70] Wetherington M. T. Nagy K. Dér L. Ábrahám Á. Noorlag J. Galajda P.. (2022). Ecological succession and the competition-colonization trade-off in microbial communities. BMC Biol. 20:262. 10.1186/s12915-022-01462-5 36447225 PMC9710175

[B71] Wickham H. (2016). Data analysis. Ggplot 2, 189–201. 10.1007/978-3-319-24277-4_9

[B72] Wilhartitz I. C. Kirschner A. K. T. Stadler H. Herndl G. J. Dietzel M. Latal C.. (2009). Heterotrophic prokaryotic production in ultraoligotrophic alpine karst aquifers and ecological implications. FEMS Microbiol. Ecol. 68, 287–299. 10.1111/j.1574-6941.2009.00679.x 19490127 PMC3119429

[B73] Wood S. N. (2010). Fast stable restricted maximum likelihood and marginal likelihood estimation of semiparametric generalized linear models. J. R. Stat. Soc. Series B: Stati. Methodol. 73, 3–36. 10.1111/j.1467-9868.2010.00749.x

[B74] Yan L. Hermans S. M. Totsche K. U. Lehmann R. Herrmann M. Küsel K. (2021). Groundwater bacterial communities evolve over time in response to recharge. Water Res. 201:117290. 10.1016/j.watres.2021.117290 34130083

[B75] Yan L. Herrmann M. Kampe B. Lehmann R. Totsche K. U. Küsel K. (2020). Environmental selection shapes the formation of near-surface groundwater microbiomes. Water Res. 170:115341. 10.1016/j.watres.2019.115341 31790889

[B76] Yilmaz P. Parfrey L. W. Yarza P. Gerken J. Pruesse E. Quast C.. (2013). The SILVA and “All-species Living Tree Project (LTP)” taxonomic frameworks. Nucleic Acids Res. 42, D643–D648. 10.1093/nar/gkt1209 24293649 PMC3965112

[B77] Zhou L. Zhang Y. Ge Y. Zhu X. Pan J. (2020). Regulatory mechanisms and promising applications of quorum sensing-inhibiting agents in control of bacterial biofilm formation. Front. Microbiol. 11:589640. 10.3389/fmicb.2020.589640 33178172 PMC7593269

[B78] Zirnstein I. Arnold T. Krawczyk-Bärsch E. Jenk U. Bernhard G. Röske I. (2012). Eukaryotic life in biofilms formed in a uranium mine. Microbiologyopen 1, 83–94. 10.1002/mbo3.17 22950016 PMC3426414

