# Supplementary material for: Primary succession of microbial communities in an aquifer from the Covey Hill formation in Quebec, Canada
Source: Front Microbiol. 2025 May 21;16:1568469. doi: 10.3389/fmicb.2025.1568469 (PMC12133808; doi:10.3389/fmicb.2025.1568469)
Supplement: Supplementary file 1 [file Data_Sheet_1.docx]

Supplementary Material

# Supplementary Figures and Tables

## Supplementary Figures


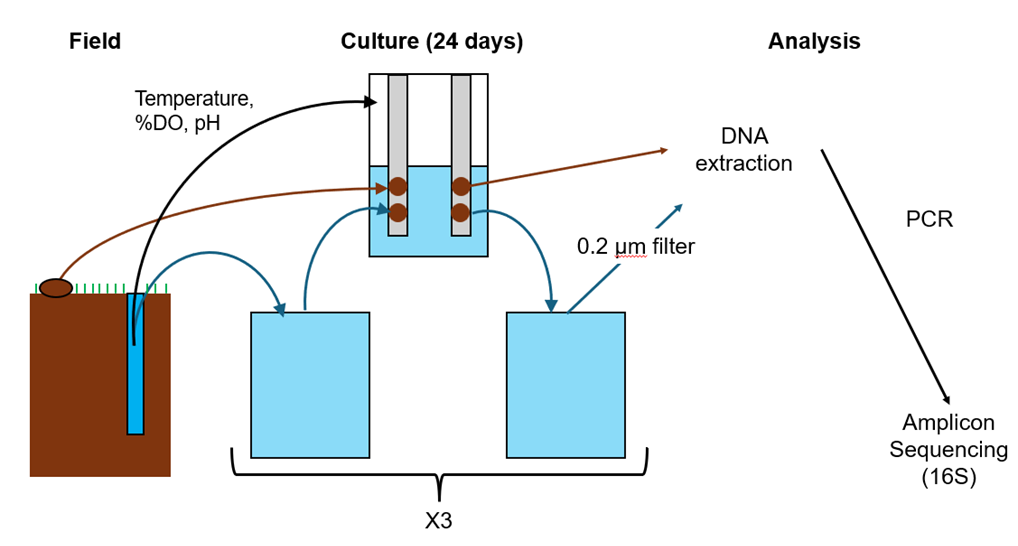


**Supplementary Figure 1.** Summary of the experimental set-up of the incubation experiment and DNA extraction. There were 24 pellets by bioreactor, but only 4 are shown for simplicity.


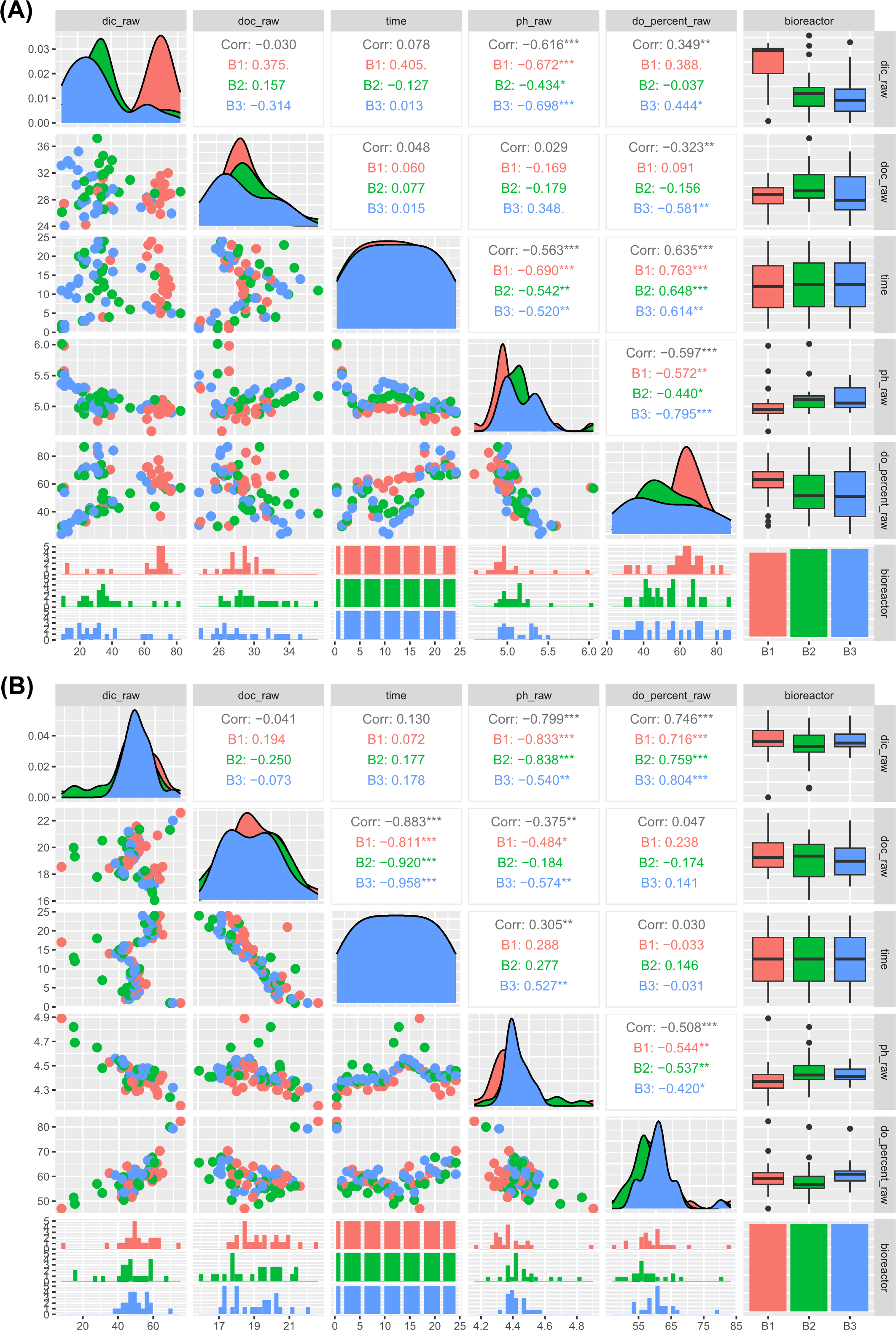


**Supplementary Figure 2.** Plots displaying Pearson correlation between the physico- and geo-chemical variables of experiment *E1* (panel A), and *E2* (panel B). Corr., Pearson correlation coefficients; dic_raw, dissolved inorganic carbon; doc_raw, dissolved organic carbon; do_percent_raw, dissolved oxygen.
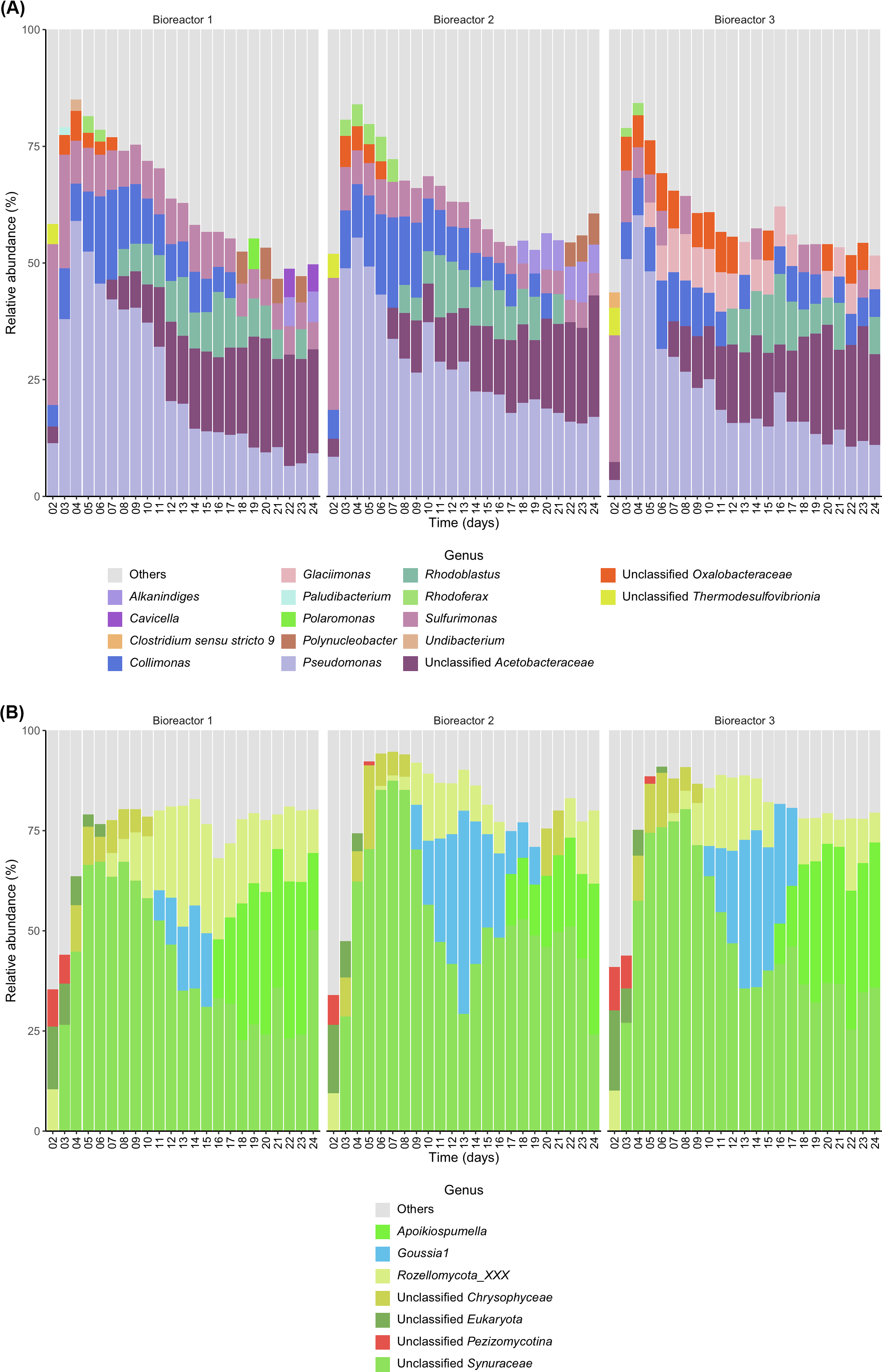


**Supplementary Figure 3.** Stacked bar chart showing the taxonomic composition of the planktonic communities of *E1* based on 16S/18S rRNA gene sequencing. Only the 5 most abundant bacterial genera and 3 most abundant eukaryote genera of each day are shown. A) Bacterial community; B) Eukaryotic community.


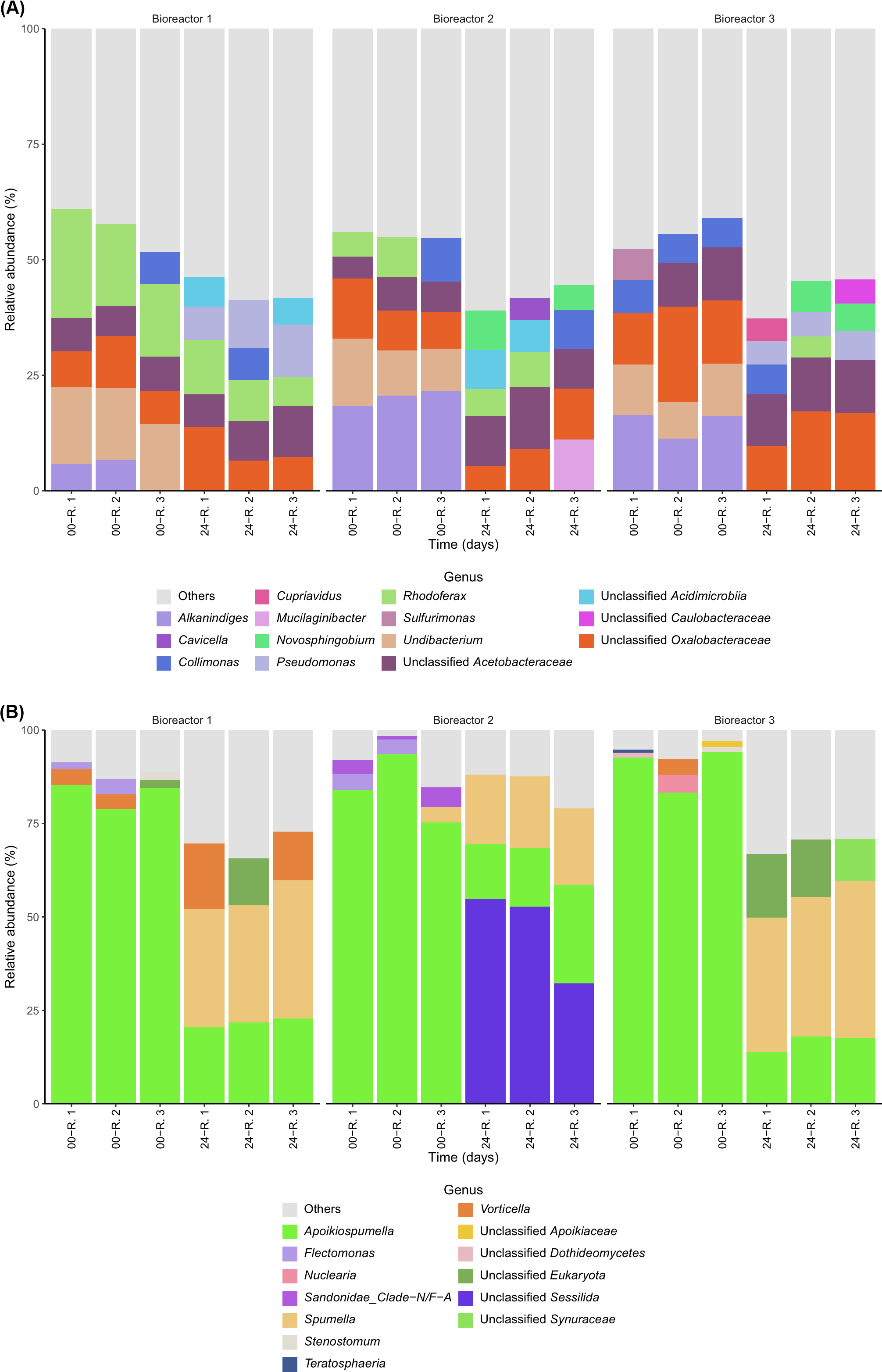


**Supplementary Figure 4.** Stacked bar chart showing the taxonomic composition of each sessile community of *E2* based on 16S/18S rRNA sequencing. Only the 5 most abundant bacterial genera and 3 most abundant eukaryote genera of each day are shown. A) Sessile bacterial community for *E2*, B) Sessile eukaryotic community for *E2*.


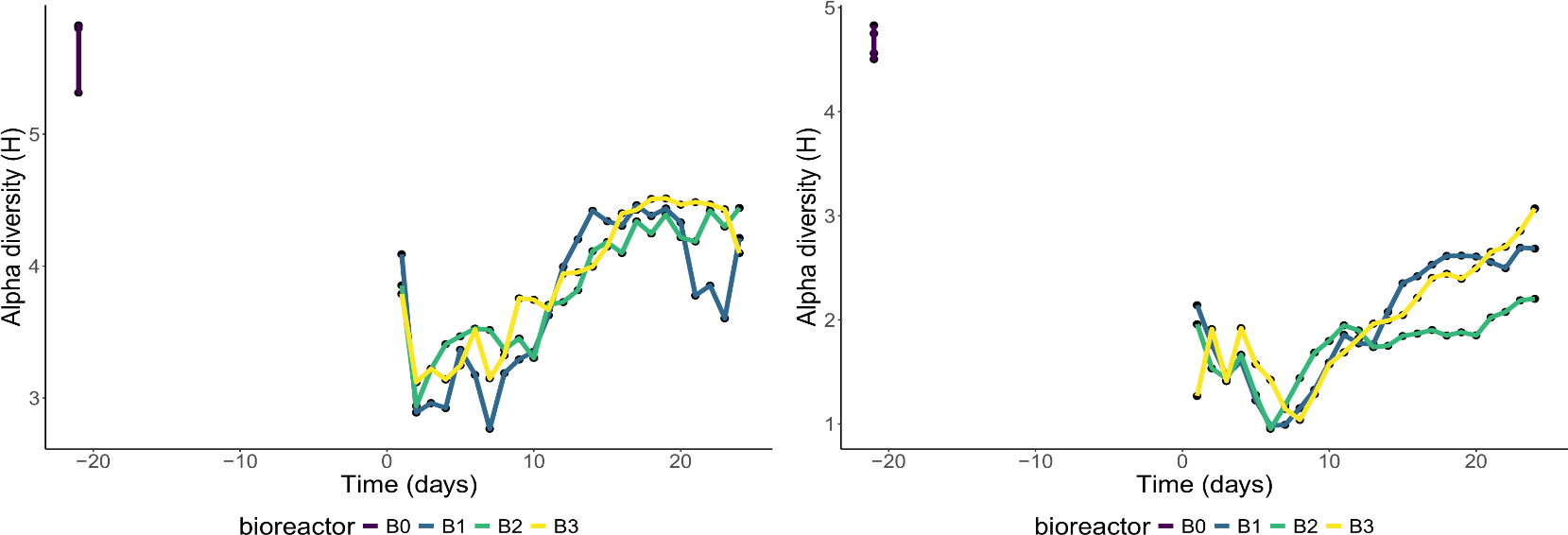

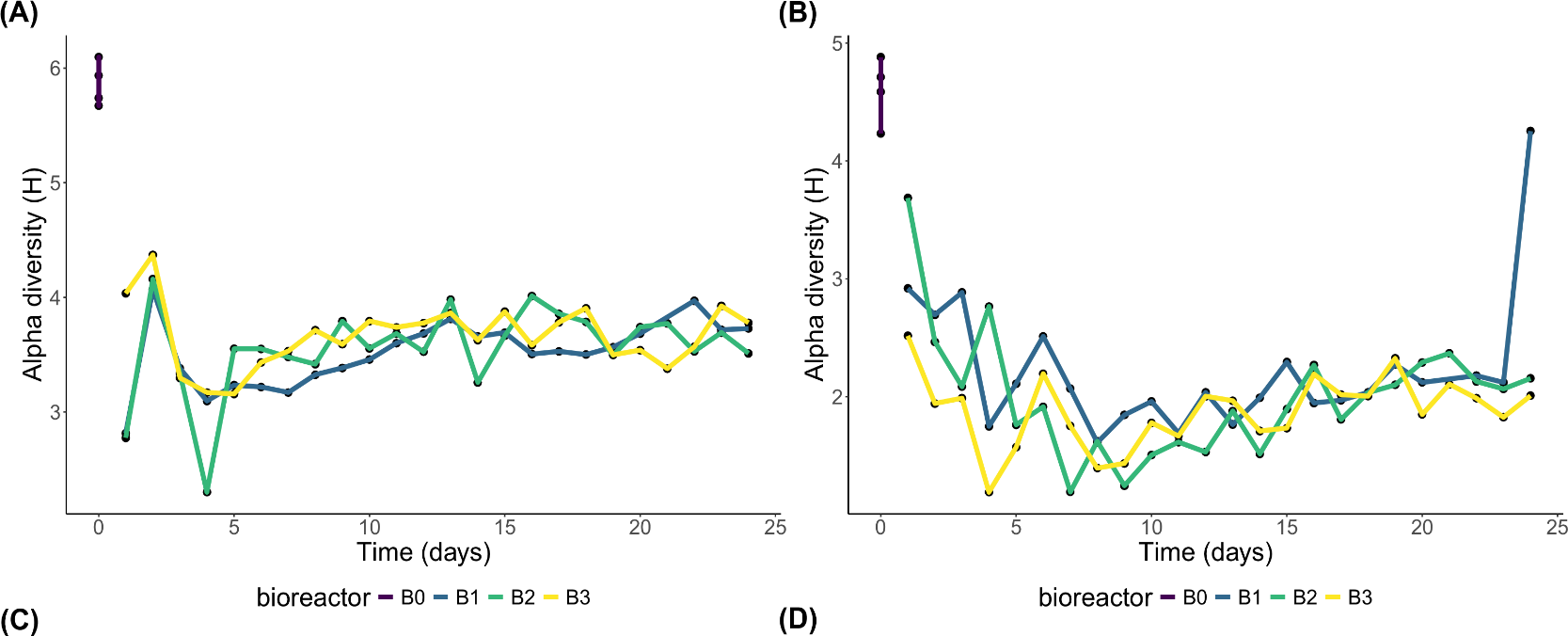


**Supplementary Figure 5.** Variation of alpha diversity (Shannon’s index) through time for each bioreactor (B1, B2, and B3) and of B0, the water collected in-situ. The gap between the in-situ sampling and the incubation for fig. C and D represent the first phase of the second experiment where no samples were collected. A) sessile bacteria experiment *E1*; B) sessile eukaryotes *E1*; C) planktonic bacteria *E2*; and D) planktonic eukaryotes *E2*.


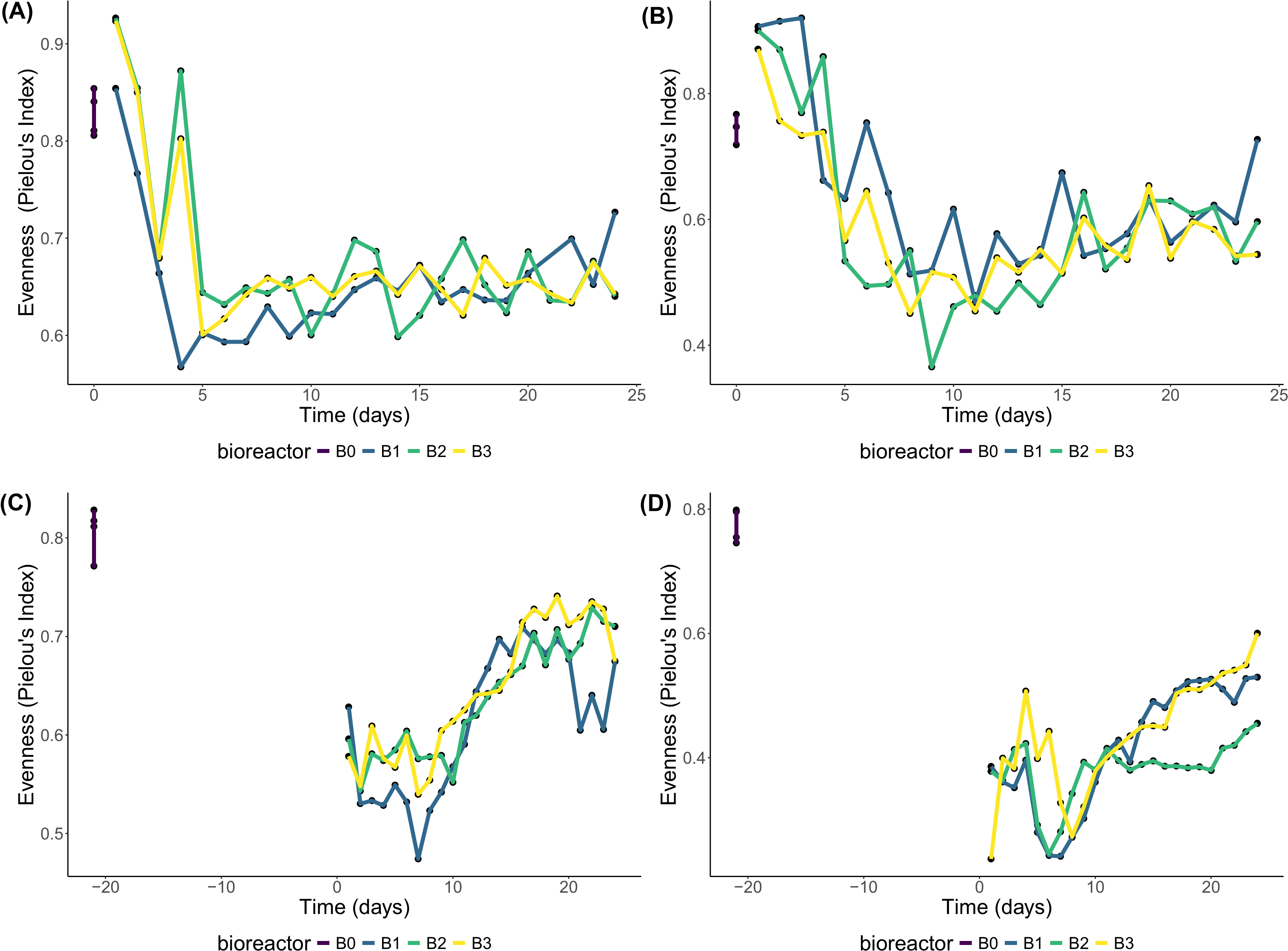


**Supplementary Figure 6.** The variation of evenness (Pielou’s Index) through time for each bioreactor (B1, B2, and B3) and of B0, the water collected in-situ. The gap between the in-situ sampling and the incubation for fig. C and D represent the first phase of the second experiment where no samples were collected. A) sessile bacteria experiment *E1*; B) sessile eukaryotes *E1*; C) planktonic bacteria *E2*; and D) planktonic eukaryotes E2.


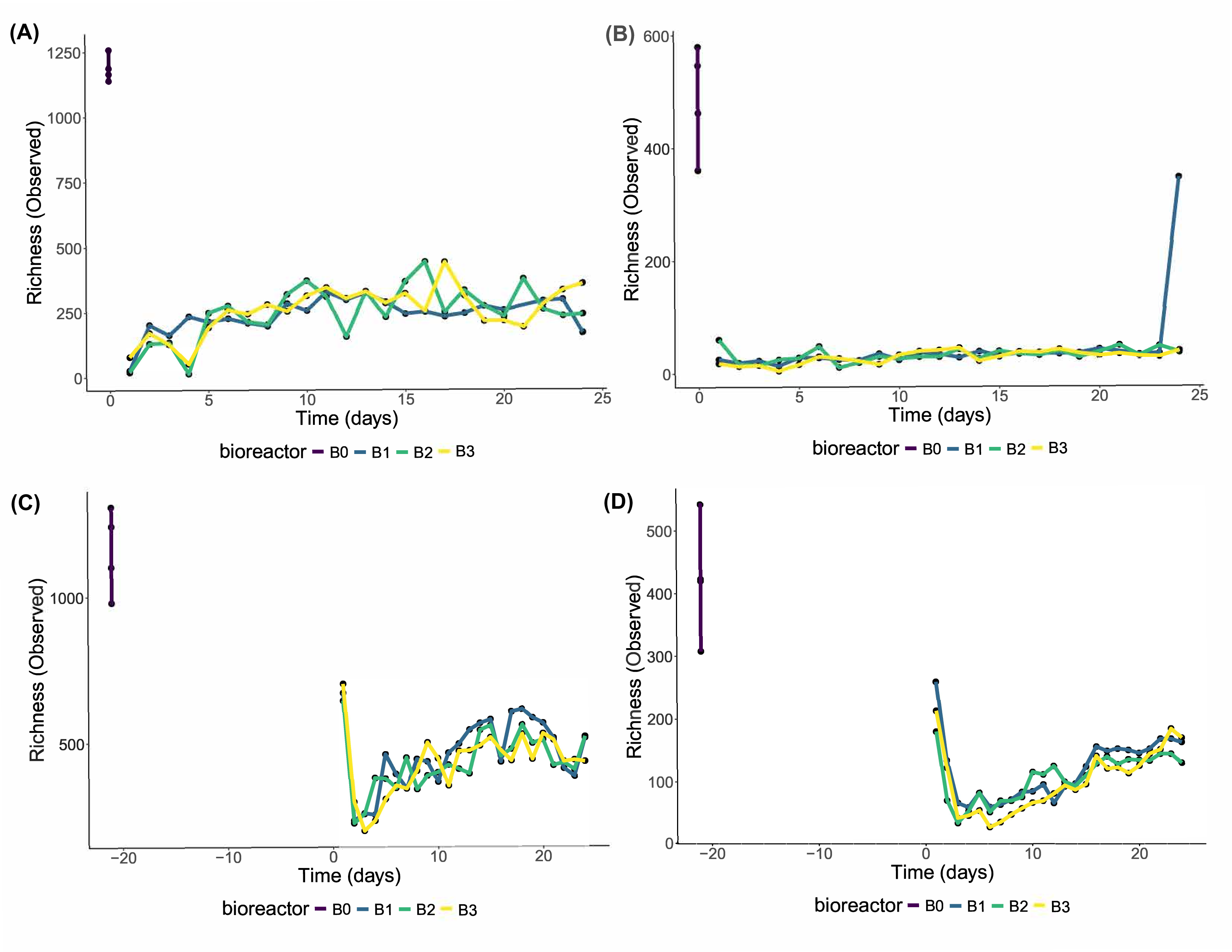


**Supplementary Figure 7.** The variation of observed richness through time for each bioreactor (B1, B2, and B3) and of B0, the water collected in-situ. The gap between the in-situ sampling and the incubation for fig. C and D represent the first phase of the second experiment where no samples were collected. A) sessile bacteria experiment *E1*; B) sessile eukaryotes *E1*; C) planktonic bacteria *E2*; and D) planktonic eukaryotes, *E2*.


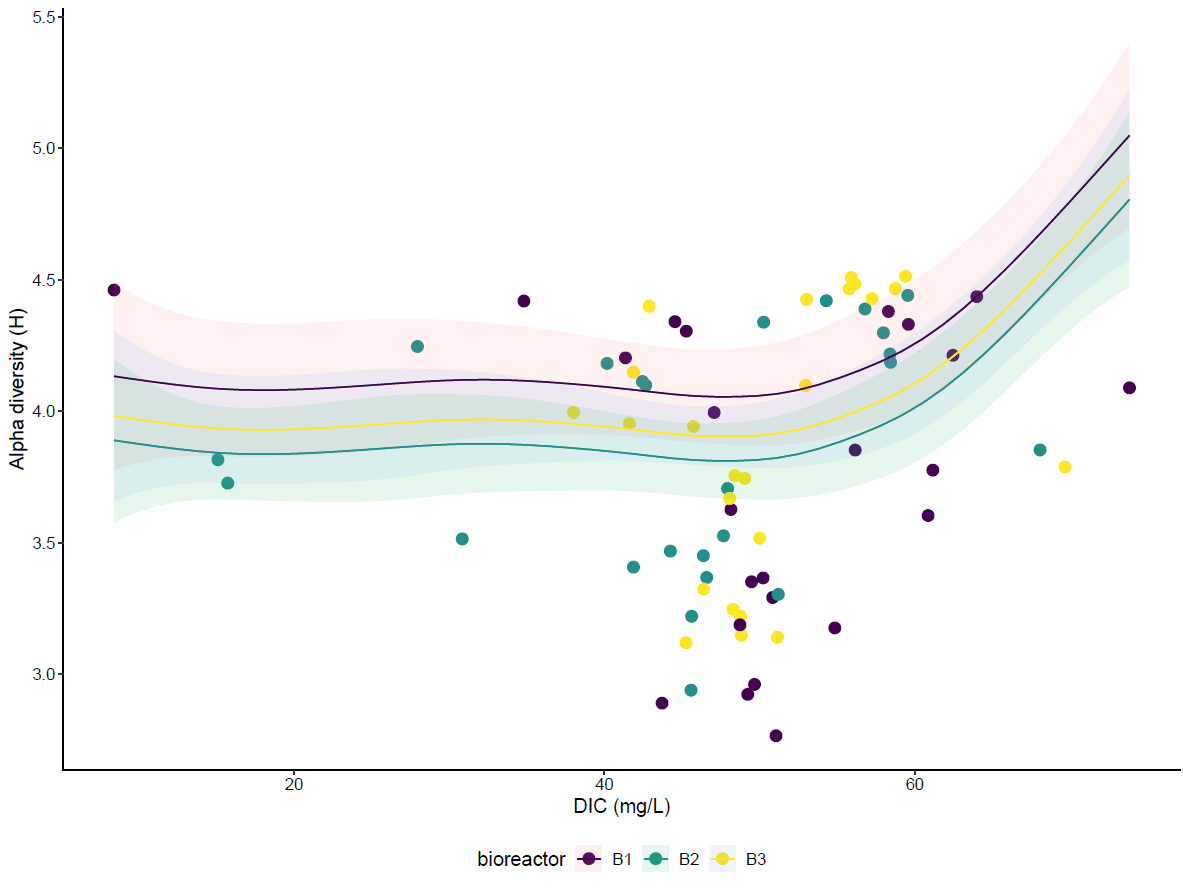


**Supplementary Figure 8.** Partial effect of DIC concentration on the Shannon index of the planktonic bacterial community of *E2*.


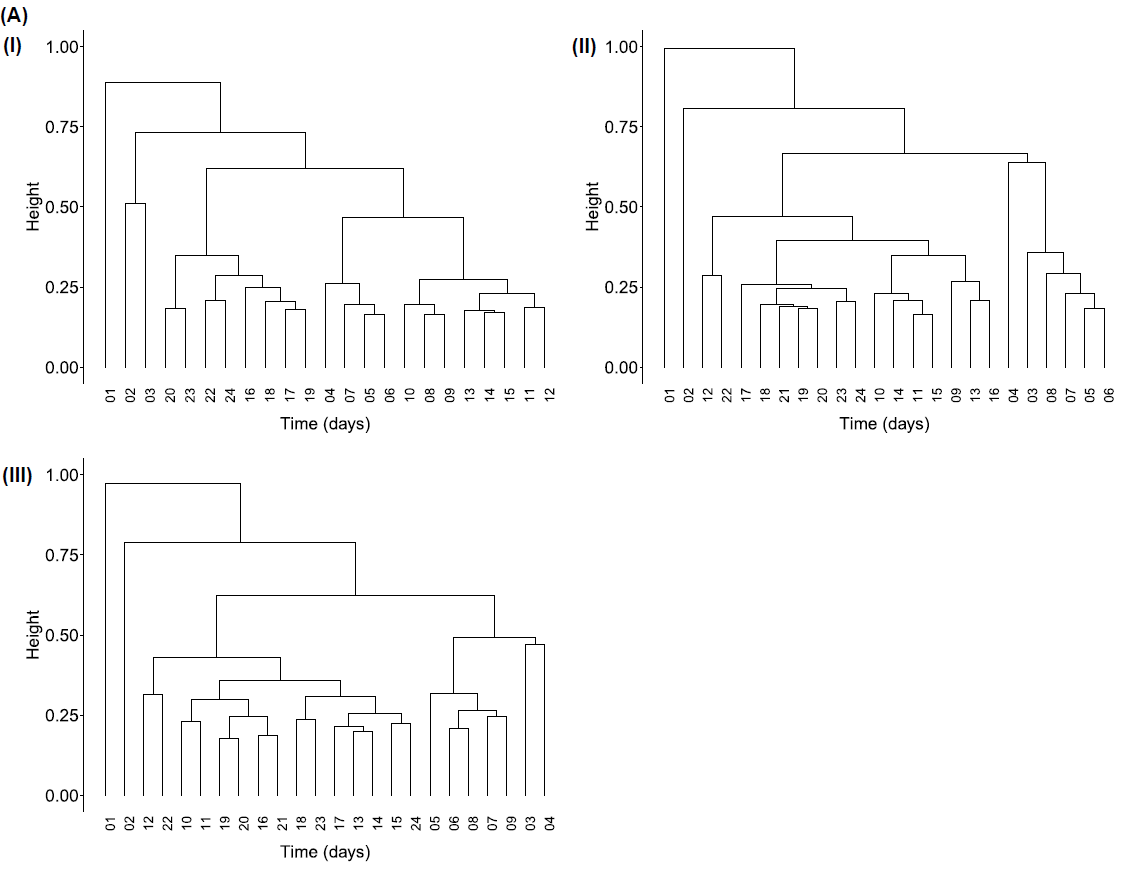


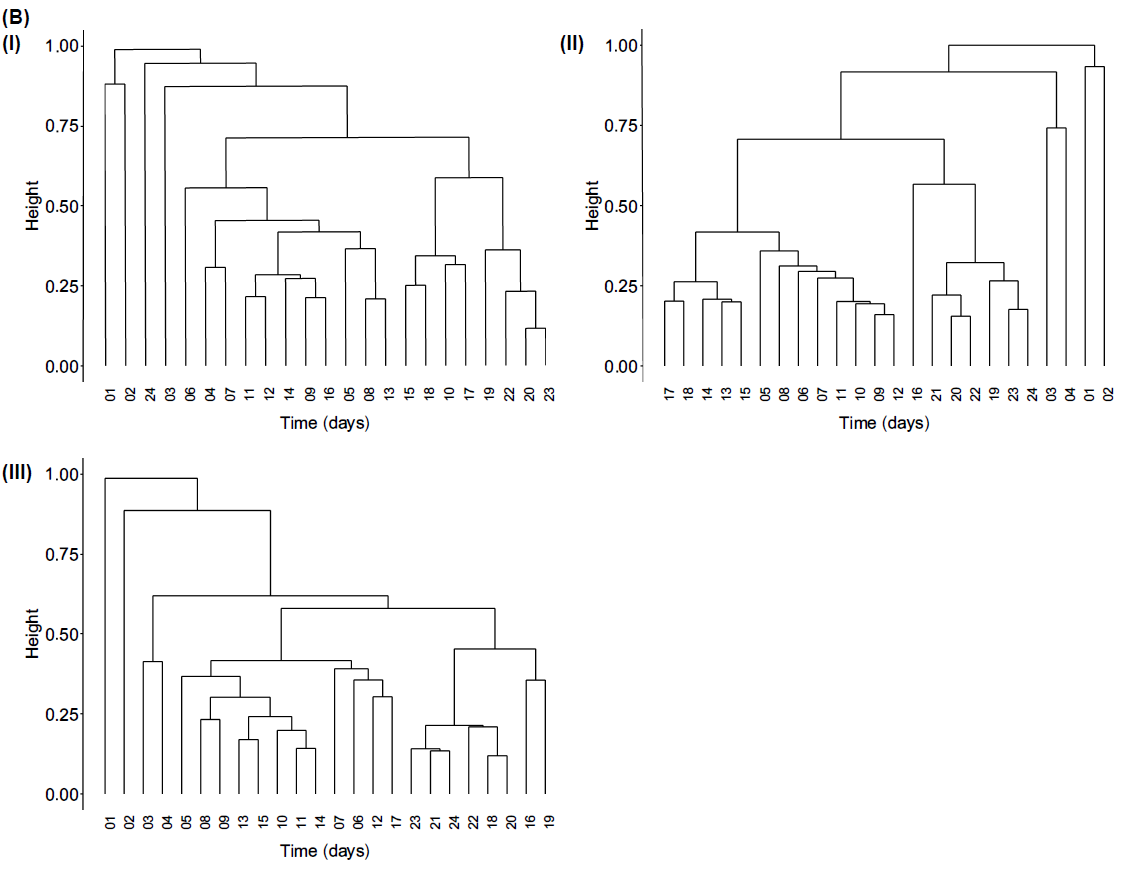


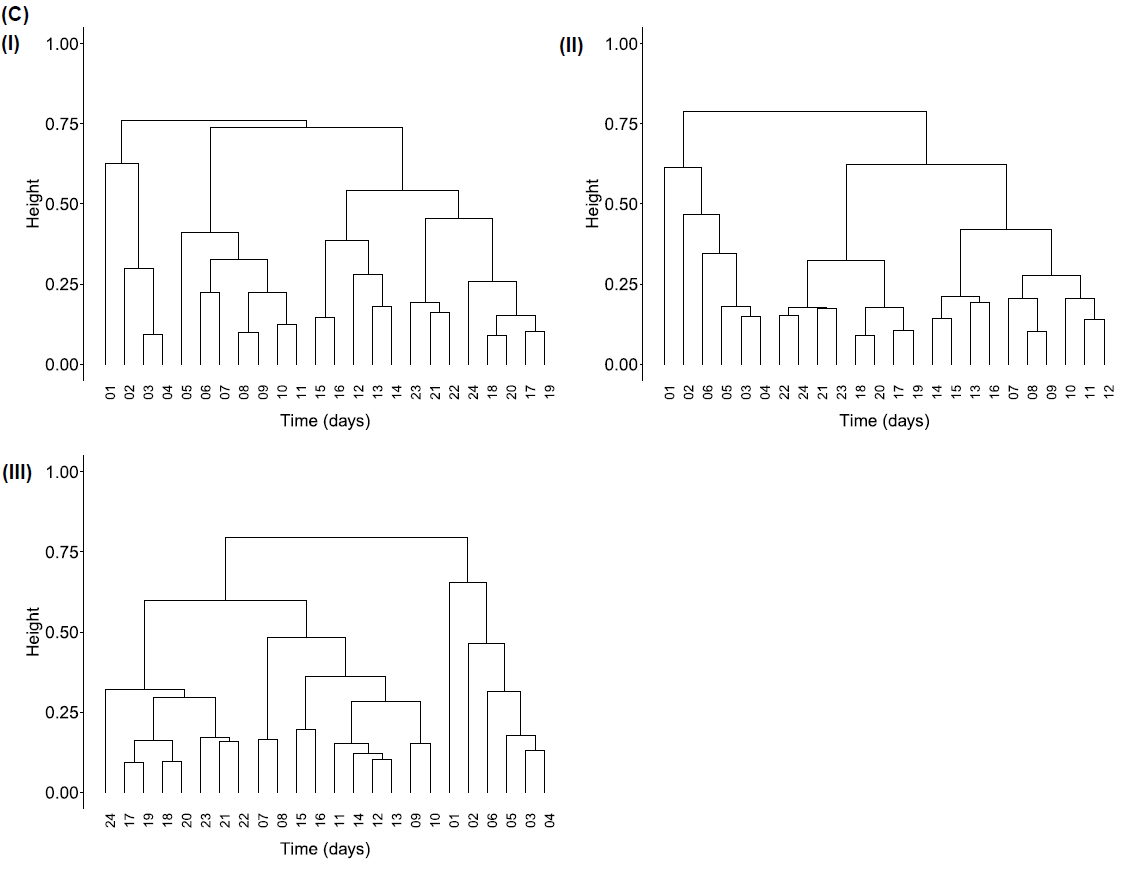


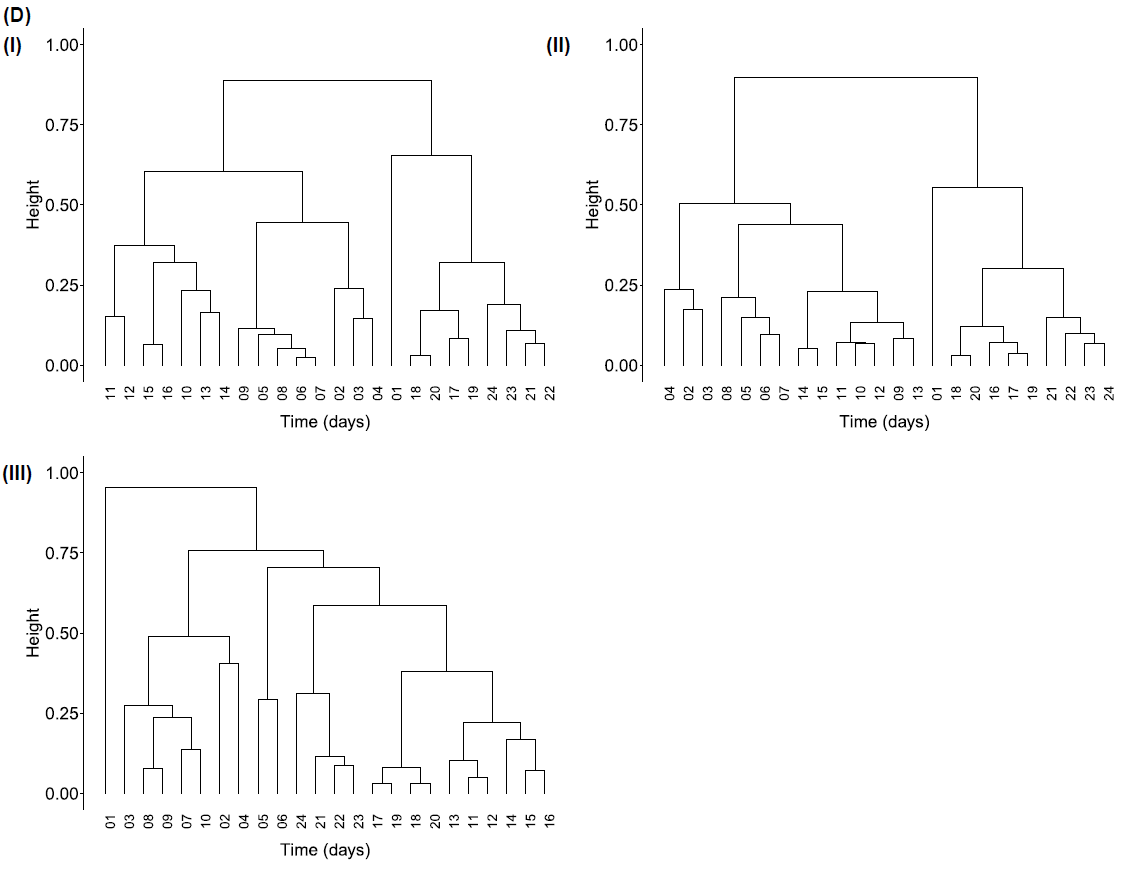


**Supplementary Figure 9.** Cluster dendrograms of the communities in each bioreactor. A) sessile bacteria experiment *E1*; B) sessile eukaryotes *E1*; C) planktonic bacteria *E2*; D) planktonic eukaryotes *E2*; I) Bioreactor B1; II) Bioreactor B2; and III) Bioreactor B3.


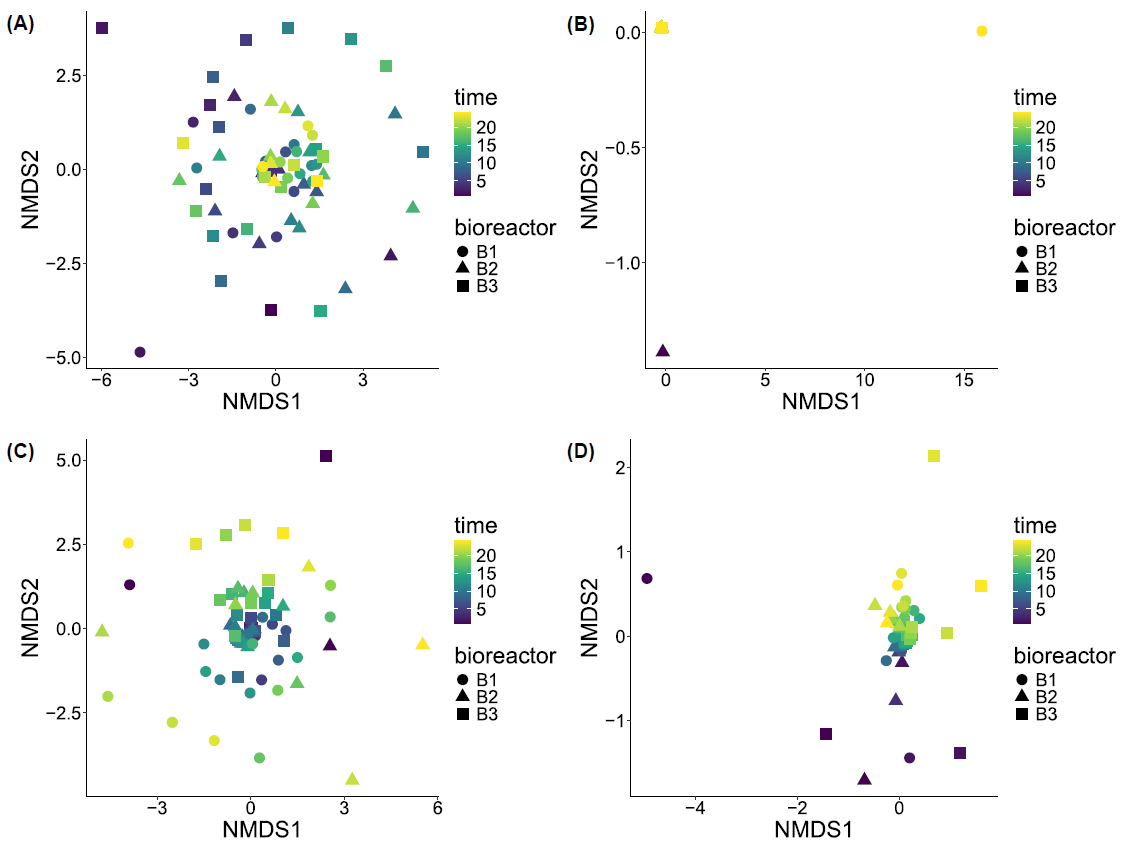


**Supplementary Figure 10.** NMDS of the beta diversity (Hellinger transformation) of all communities. A) sessile bacteria *E1*, stress = 0.0905; B) sessile eukaryotes *E1*, stress **=** 8.56*10^-5; C) planktonic bacteria, *E2*, stress = 0.1079; D) planktonic eukaryotes, *E2*, stress = 0.114.


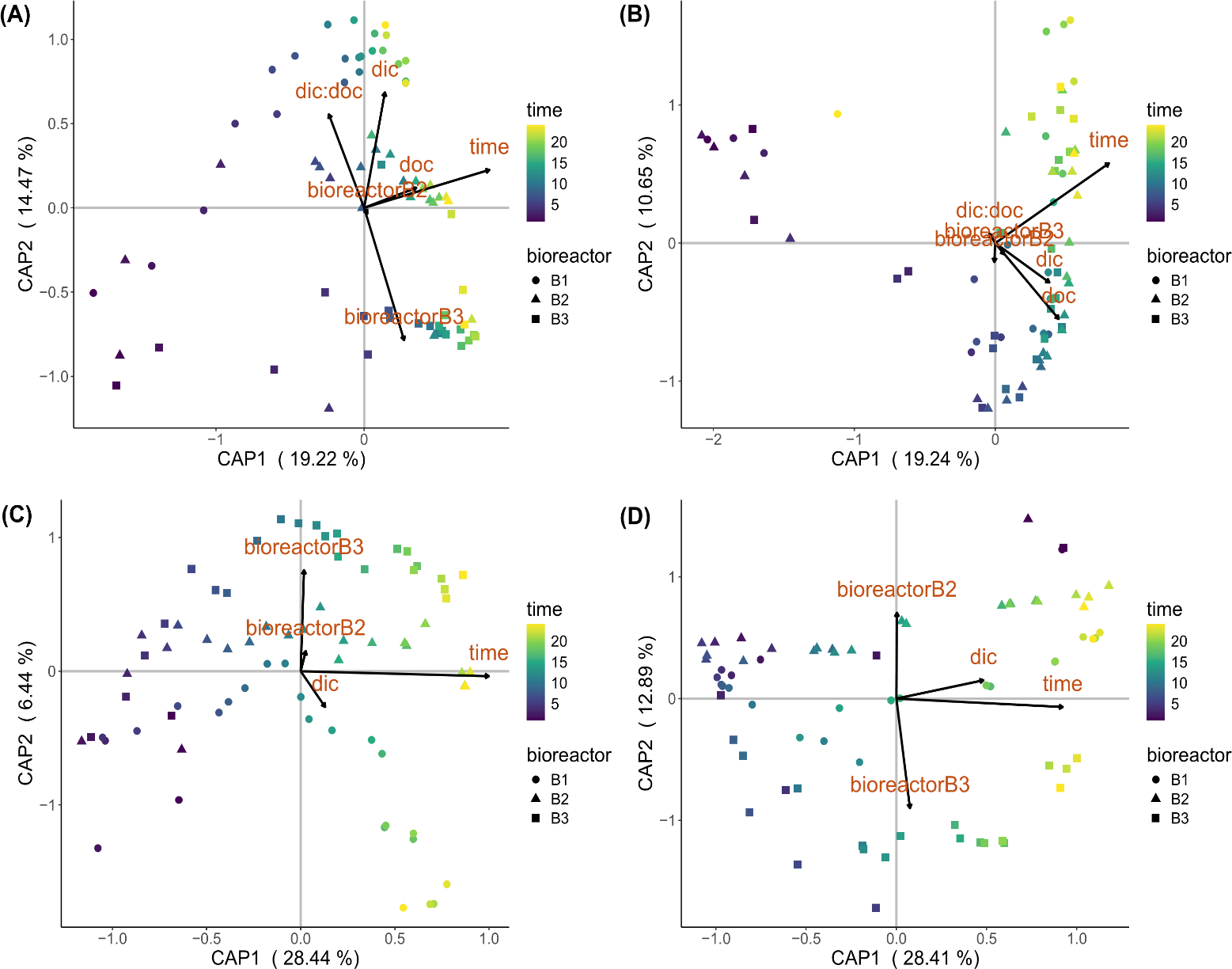


**Supplementary Figure 11.** Db-RDA graph showing the correlation of physico- and geo-chemical parameters (DIC, DOC, interaction of DIC and DOC, time and each individual bioreactor) with the beta diversity of all communities (Bray-Curtis matrix) through time. A) sessile bacteria experiment *E1*; B) sessile eukaryotes *E1*; C) planktonic bacteria *E2*; and D) planktonic eukaryotes E2. DOC, dissolved organic carbon; DIC, dissolved inorganic carbon. B1 is not displayed as B2 and B3 are compared to B1.


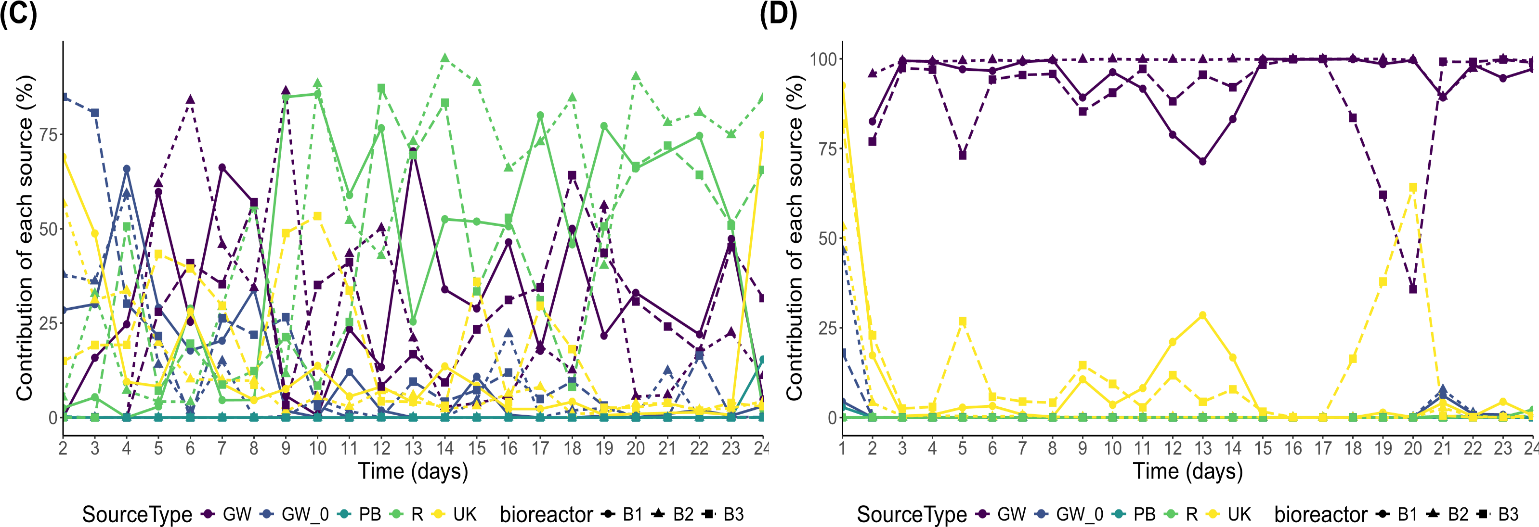

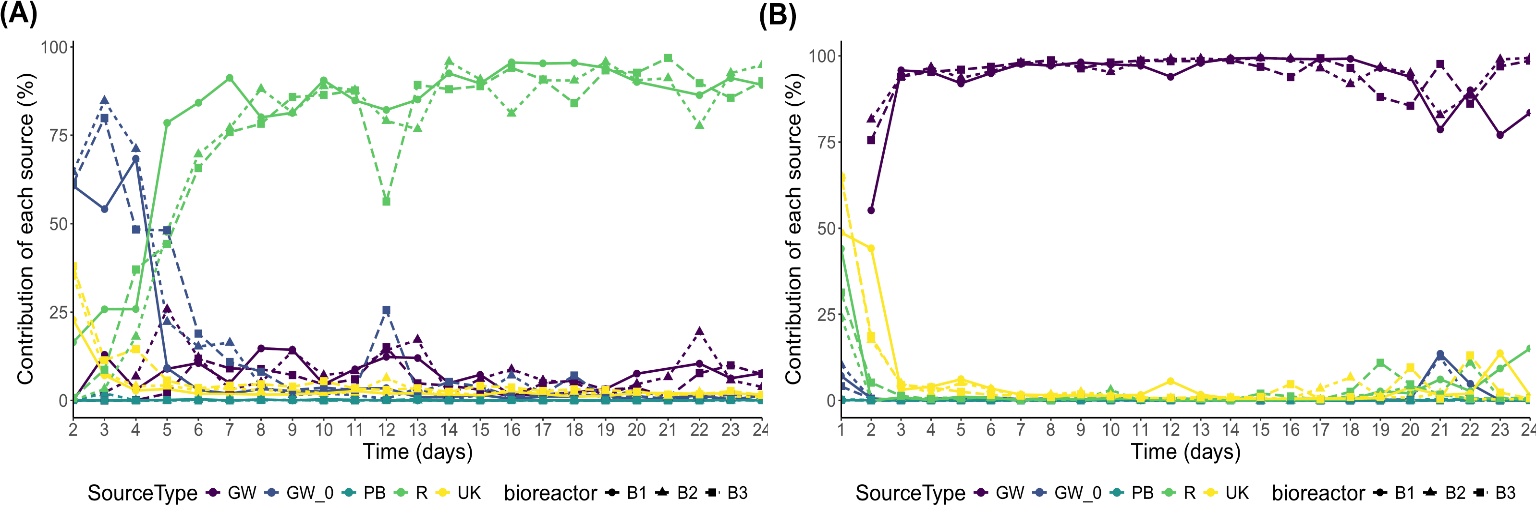


**Supplementary Figure 12.** Summed cumulative contribution percentages of each source through time for A) sessile community of Bacteria experiment *E1*; B) planktonic community of bacteria *E2*; C); The sessile community of Eukaryotes *E1*; and D) planktonic community of Eukaryotes *E2*. GW, planktonic community in the incubation water; GW_0, planktonic community in the groundwater collected at the sampling site; PB, community of the peat bog at the sampling site; R, sessile community; and UK, remainder of the variation that is attributed to an unknown source.

## Supplementary Tables

**Supplementary Table 1**. PCR conditions used for 16S or 18S rRNA gene amplification.

| Domain |  | Denaturation | Annealing | Extension | Final Extension |
| --- | --- | --- | --- | --- | --- |
| Bacteria | Nb of cycles |  | 35 |  | 1 |
|  | Time (s) | 30 | 60 |  | 30 |
|  | Temperature (˚C) | 98 | 57 |  | 98 |
| Eukaryote | Nb of cycles |  | 35 |  | 1 |
|  | Time (s) | 30 | 30 |  | 30 |
|  | Temperature (˚C) | 98 | 61 |  | 98 |
| Archaea | Nb of cycles |  | 35 |  | 1 |
|  | Time (s) | 30 | 30 | 60 | 600 |
|  | Temperature (˚C) | 98 | 67 | 72 | 72 |

**Supplementary Table 2.** Summary of the geochemical characteristics of each experiment: A) *E1*, B) *E2*; and C) Raw geochemical measurements for all samples.

| A) |  |  |  |  |
| --- | --- | --- | --- | --- |
|  | pH | Dissolved O_2_ (%) | DIC (mg/L) | DOC (mg/L) |
| Min. | 4.600 | 23.900 | 10.350 | 24.110 |
| 1st Qu. | 4.955 | 42.000 | 24.090 | 27.320 |
| Median | 5.030 | 56.800 | 35.320 | 28.900 |
| Mean | 5.096 | 55.340 | 41.780 | 29.170 |
| 3rd Qu. | 5.190 | 66.900 | 65.260 | 30.870 |
| Max. | 6.010 | 86.800 | 81.680 | 37.210 |
| B) |  |  |  |  |
|  | pH | Dissolved O_2_ (%) | DIC (mg/L) | DOC (mg/L) |
| Min. | 4.170 | 47.000 | 8.410 | 16.060 |
| 1st Qu. | 4.370 | 56.250 | 45.090 | 18.060 |
| Median | 4.410 | 58.850 | 48.940 | 19.230 |
| Mean | 4.425 | 59.700 | 48.930 | 19.160 |
| 3rd Qu. | 4.480 | 61.850 | 56.150 | 20.250 |
| Max. | 4.890 | 82.300 | 73.830 | 22.590 |

C)

| sample name | Sample | Rock pellet position | Temperature (˚C) | pH | Dissolved O_2_ (%) | DIC (mg/L) | DOC (mg/L) |
| --- | --- | --- | --- | --- | --- | --- | --- |
|  | type |  |  |  |  |  |  |
| E1-R-T01-B1-1 | Rock | 1 | 13-14 | 5.98 | 57.5 |  |  |
| E1-R-T01-B2-1 | Rock | 1 | 13-14 | 6.01 | 56.8 |  |  |
| E1-R-T01-B3-1 | Rock | 1 | 13-14 | 5.33 | 53.9 |  |  |
| E1-R-T02-B1-1 | Rock | 2 | 13-14 | 5.57 | 29.9 | 11.85 | 27.405 |
| E1-R-T02-B2-1 | Rock | 2 | 13-14 | 5.53 | 29.5 | 10.62 | 26.145 |
| E1-R-T02-B3-1 | Rock | 2 | 13-14 | 5.51 | 30.4 | 12.215 | 24.105 |
| E1-R-T03-B1-1 | Rock | 3 | 13-14 | 5.29 | 32.9 | 24.845 | 24.265 |
| E1-R-T03-B2-1 | Rock | 3 | 13-14 | 5.24 | 33.6 | 22.965 | 28.005 |
| E1-R-T03-B3-1 | Rock | 3 | 13-14 | 5.31 | 36.8 | 24 | 25.415 |
| E1-R-T04-B1-1 | Rock | 1 | 13-14 | 5.12 | 43.5 | 35.11 | 31.25 |
| E1-R-T04-B2-1 | Rock | 1 | 13-14 | 5.05 | 47.7 | 38.83 | 32.53 |
| E1-R-T04-B3-1 | Rock | 1 | 13-14 | 5.14 | 42.8 | 27.495 | 31.435 |
| E1-R-T05-B1-1 | Rock | 2 | 13-14 | 4.88 | 61.4 | 69.61 | 25.855 |
| E1-R-T05-B2-1 | Rock | 2 | 13-14 | 4.93 | 57.3 | 81.68 | 29.215 |
| E1-R-T05-B3-1 | Rock | 2 | 13-14 | 4.9 | 56.4 | 76.255 | 25.945 |
| E1-R-T06-B1-1 | Rock | 3 | 13-14 | 4.94 | 58.6 | 70.925 | 29.225 |
| E1-R-T06-B2-1 | Rock | 3 | 13-14 | 4.98 | 53.3 | 51.485 | 30.97 |
| E1-R-T06-B3-1 | Rock | 3 | 13-14 | 5.04 | 55.8 | 64.275 | 27.96 |
| E1-R-T07-B1-1 | Rock | 1 | 13-14 | 4.9 | 63.4 | 69.6 | 28.795 |
| E1-R-T07-B2-1 | Rock | 1 | 13-14 | 5.03 | 56.1 | 73.305 | 28.635 |
| E1-R-T07-B3-1 | Rock | 1 | 13-14 | 5.02 | 61.5 | 61.44 | 28.35 |
| E1-R-T08-B1-1 | Rock | 2 | 13-14 | 5.08 | 36.8 | 69.675 | 31.58 |
| E1-R-T08-B2-1 | Rock | 2 | 13-14 | 5.13 | 42.8 | 44.685 | 29.12 |
| E1-R-T08-B3-1 | Rock | 2 | 13-14 | 4.99 | 47.8 | 36.86 | 29.01 |
| E1-R-T09-B1-1 | Rock | 3 | 13-14 | 4.97 | 60.4 | 66.505 | 30.46 |
| E1-R-T09-B2-1 | Rock | 3 | 13-14 | 5.12 | 41.2 | 27.3 | 29.42 |
| E1-R-T09-B3-1 | Rock | 3 | 13-14 | 5.28 | 35.9 | 19.305 | 30.235 |
| E1-R-T10-B1-1 | Rock | 1 | 13-14 | 5.01 | 57.1 | 73.745 | 28.94 |
| E1-R-T10-B2-1 | Rock | 1 | 13-14 | 5.13 | 45.5 | 40.6 | 33.93 |
| E1-R-T10-B3-1 | Rock | 1 | 13-14 | 5.4 | 25.8 | 14.215 | 33.82 |
| E1-R-T11-B1-1 | Rock | 2 | 13-14 | 4.96 | 62.4 | 71.205 | 29.07 |
| E1-R-T11-B2-1 | Rock | 2 | 13-14 | 5.17 | 38.8 | 31.755 | 37.21 |
| E1-R-T11-B3-1 | Rock | 2 | 13-14 | 5.37 | 23.9 | 10.345 | 33.135 |
| E1-R-T12-B1-1 | Rock | 3 | 13-14 | 4.97 | 54.9 | 75.71 | 28.9 |
| E1-R-T12-B2-1 | Rock | 3 | 13-14 | 5.21 | 40.2 | 35.765 | 31.88 |
| E1-R-T12-B3-1 | Rock | 3 | 13-14 | 5.35 | 32.1 | 15.775 | 33.305 |
| E1-R-T13-B1-1 | Rock | 1 | 13-14 | 5.11 | 63.7 | 70.91 | 28.73 |
| E1-R-T13-B2-1 | Rock | 1 | 13-14 | 5.16 | 48.8 | 35.505 | 29.765 |
| E1-R-T13-B3-1 | Rock | 1 | 13-14 | 5.3 | 34.2 | 20.155 | 32.435 |
| E1-R-T14-B1-1 | Rock | 2 | 13-14 | 4.91 | 65.5 | 74.165 | 32.01 |
| E1-R-T14-B2-1 | Rock | 2 | 13-14 | 5.14 | 41.2 | 32.28 | 29.82 |
| E1-R-T14-B3-1 | Rock | 2 | 13-14 | 5.3 | 38.4 | 20.525 | 35.245 |
| E1-R-T15-B1-1 | Rock | 3 | 13-14 | 4.94 | 64.3 | 72.075 | 30.35 |
| E1-R-T15-B2-1 | Rock | 3 | 13-14 | 5.11 | 43.8 | 66.23 | 28.965 |
| E1-R-T15-B3-1 | Rock | 3 | 13-14 | 4.98 | 40.1 | 29.625 | 31.555 |
| E1-R-T16-B1-1 | Rock | 1 | 13-14 | 4.93 | 64.7 | 72.365 | 28.5 |
| E1-R-T16-B2-1 | Rock | 1 | 13-14 | 5.23 | 49.2 | 34.48 | 31.72 |
| E1-R-T16-B3-1 | Rock | 1 | 13-14 | 5.21 | 47.4 | 41.105 | 29.625 |
| E1-R-T17-B1-1 | Rock | 2 | 13-14 | 4.97 | 70.4 | 69.1 | 27.4 |
| E1-R-T17-B2-1 | Rock | 2 | 13-14 | 5.17 | 53.7 | 35.32 | 34.59 |
| E1-R-T17-B3-1 | Rock | 2 | 13-14 | 4.96 | 65.8 | 58.075 | 26.855 |
| E1-R-T18-B1-1 | Rock | 3 | 13-14 | 4.77 | 82.6 | 32.045 | 29.26 |
| E1-R-T18-B2-1 | Rock | 3 | 13-14 | 4.88 | 86.8 | 23.98 | 30.765 |
| E1-R-T18-B3-1 | Rock | 3 | 13-14 | 4.99 | 86.7 | 31.94 | 27.24 |
| E1-R-T19-B1-1 | Rock | 1 | 13-14 | 4.86 | 70.4 | 39.62 | 26.985 |
| E1-R-T19-B2-1 | Rock | 1 | 13-14 | 4.96 | 70.7 | 23.295 | 27.92 |
| E1-R-T19-B3-1 | Rock | 1 | 13-14 | 4.98 | 72 | 25.165 | 27.93 |
| E1-R-T20-B1-1 | Rock | 2 | 13-14 | 4.8 | 72 | 61.54 | 28.145 |
| E1-R-T20-B2-1 | Rock | 2 | 13-14 | 4.98 | 66.5 | 20.68 | 27.23 |
| E1-R-T20-B3-1 | Rock | 2 | 13-14 | 5.07 | 69.5 | 22.7 | 27.535 |
| E1-R-T21-B2-1 | Rock | 3 | 13-14 | 5 | 66.1 | 24.17 | 28.355 |
| E1-R-T21-B3-1 | Rock | 3 | 13-14 | 4.97 | 84.1 | 41.435 | 26.515 |
| E1-R-T22-B1-1 | Rock | 1 | 13-14 | 4.88 | 77.2 | 68.485 | 30.38 |
| E1-R-T22-B2-1 | Rock | 1 | 13-14 | 4.98 | 73.8 | 34.175 | 28.44 |
| E1-R-T22-B3-1 | Rock | 1 | 13-14 | 5.04 | 71.9 | 30.34 | 25.935 |
| E1-R-T23-B1-1 | Rock | 2 | 13-14 | 4.95 | 72.1 | 62.315 | 27.675 |
| E1-R-T23-B2-1 | Rock | 2 | 13-14 | 5.08 | 66.7 | 32.875 | 33.46 |
| E1-R-T23-B3-1 | Rock | 2 | 13-14 | 4.93 | 80.7 | 32.845 | 26.425 |
| E1-R-T24-B1-1 | Rock | 3 | 13-14 | 4.6 | 67.1 | 64.285 | 26.61 |
| E1-R-T24-B2-1 | Rock | 3 | 13-14 | 4.89 | 67.5 | 35.715 | 26.55 |
| E1-R-T24-B3-1 | Rock | 3 | 13-14 | 4.92 | 68.5 | 32.44 | 26.65 |
| E1-W-T02-B1-1 | Groundwater | 2 | 13-14 | 5.57 | 29.9 | 11.85 | 27.405 |
| E1-W-T02-B2-1 | Groundwater | 2 | 13-14 | 5.53 | 29.5 | 10.62 | 26.145 |
| E1-W-T02-B3-1 | Groundwater | 2 | 13-14 | 5.51 | 30.4 | 12.215 | 24.105 |
| E1-W-T03-B1-1 | Groundwater | 3 | 13-14 | 5.29 | 32.9 | 24.845 | 24.265 |
| E1-W-T03-B2-1 | Groundwater | 3 | 13-14 | 5.24 | 33.6 | 22.965 | 28.005 |
| E1-W-T03-B3-1 | Groundwater | 3 | 13-14 | 5.31 | 36.8 | 24 | 25.415 |
| E1-W-T04-B1-1 | Groundwater | 1 | 13-14 | 5.12 | 43.5 | 35.11 | 31.25 |
| E1-W-T04-B2-1 | Groundwater | 1 | 13-14 | 5.05 | 47.7 | 38.83 | 32.53 |
| E1-W-T04-B3-1 | Groundwater | 1 | 13-14 | 5.14 | 42.8 | 27.495 | 31.435 |
| E1-W-T05-B1-1 | Groundwater | 2 | 13-14 | 4.88 | 61.4 | 69.61 | 25.855 |
| E1-W-T05-B2-1 | Groundwater | 2 | 13-14 | 4.93 | 57.3 | 81.68 | 29.215 |
| E1-W-T05-B3-1 | Groundwater | 2 | 13-14 | 4.9 | 56.4 | 76.255 | 25.945 |
| E1-W-T06-B1-1 | Groundwater | 3 | 13-14 | 4.94 | 58.6 | 70.925 | 29.225 |
| E1-W-T06-B2-1 | Groundwater | 3 | 13-14 | 4.98 | 53.3 | 51.485 | 30.97 |
| E1-W-T06-B3-1 | Groundwater | 3 | 13-14 | 5.04 | 55.8 | 64.275 | 27.96 |
| E1-W-T07-B1-1 | Groundwater | 1 | 13-14 | 4.9 | 63.4 | 69.6 | 28.795 |
| E1-W-T07-B2-1 | Groundwater | 1 | 13-14 | 5.03 | 56.1 | 73.305 | 28.635 |
| E1-W-T07-B3-1 | Groundwater | 1 | 13-14 | 5.02 | 61.5 | 61.44 | 28.35 |
| E1-W-T08-B1-1 | Groundwater | 2 | 13-14 | 5.08 | 36.8 | 69.675 | 31.58 |
| E1-W-T08-B2-1 | Groundwater | 2 | 13-14 | 5.13 | 42.8 | 44.685 | 29.12 |
| E1-W-T08-B3-1 | Groundwater | 2 | 13-14 | 4.99 | 47.8 | 36.86 | 29.01 |
| E1-W-T09-B1-1 | Groundwater | 3 | 13-14 | 4.97 | 60.4 | 66.505 | 30.46 |
| E1-W-T09-B2-1 | Groundwater | 3 | 13-14 | 5.12 | 41.2 | 27.3 | 29.42 |
| E1-W-T09-B3-1 | Groundwater | 3 | 13-14 | 5.28 | 35.9 | 19.305 | 30.235 |
| E1-W-T10-B1-1 | Groundwater | 1 | 13-14 | 5.01 | 57.1 | 73.745 | 28.94 |
| E1-W-T10-B2-1 | Groundwater | 1 | 13-14 | 5.13 | 45.5 | 40.6 | 33.93 |
| E1-W-T10-B3-1 | Groundwater | 1 | 13-14 | 5.4 | 25.8 | 14.215 | 33.82 |
| E1-W-T11-B1-1 | Groundwater | 2 | 13-14 | 4.96 | 62.4 | 71.205 | 29.07 |
| E1-W-T11-B2-1 | Groundwater | 2 | 13-14 | 5.17 | 38.8 | 31.755 | 37.21 |
| E1-W-T11-B3-1 | Groundwater | 2 | 13-14 | 5.37 | 23.9 | 10.345 | 33.135 |
| E1-W-T12-B1-1 | Groundwater | 3 | 13-14 | 4.97 | 54.9 | 75.71 | 28.9 |
| E1-W-T12-B2-1 | Groundwater | 3 | 13-14 | 5.21 | 40.2 | 35.765 | 31.88 |
| E1-W-T12-B3-1 | Groundwater | 3 | 13-14 | 5.35 | 32.1 | 15.775 | 33.305 |
| E1-W-T13-B1-1 | Groundwater | 1 | 13-14 | 5.11 | 63.7 | 70.91 | 28.73 |
| E1-W-T13-B2-1 | Groundwater | 1 | 13-14 | 5.16 | 48.8 | 35.505 | 29.765 |
| E1-W-T13-B3-1 | Groundwater | 1 | 13-14 | 5.3 | 34.2 | 20.155 | 32.435 |
| E1-W-T14-B1-1 | Groundwater | 2 | 13-14 | 4.91 | 65.5 | 74.165 | 32.01 |
| E1-W-T14-B2-1 | Groundwater | 2 | 13-14 | 5.14 | 41.2 | 32.28 | 29.82 |
| E1-W-T14-B3-1 | Groundwater | 2 | 13-14 | 5.3 | 38.4 | 20.525 | 35.245 |
| E1-W-T15-B1-1 | Groundwater | 3 | 13-14 | 4.94 | 64.3 | 72.075 | 30.35 |
| E1-W-T15-B2-1 | Groundwater | 3 | 13-14 | 5.11 | 43.8 | 66.23 | 28.965 |
| E1-W-T15-B3-1 | Groundwater | 3 | 13-14 | 4.98 | 40.1 | 29.625 | 31.555 |
| E1-W-T16-B1-1 | Groundwater | 1 | 13-14 | 4.93 | 64.7 | 72.365 | 28.5 |
| E1-W-T16-B2-1 | Groundwater | 1 | 13-14 | 5.23 | 49.2 | 34.48 | 31.72 |
| E1-W-T16-B3-1 | Groundwater | 1 | 13-14 | 5.21 | 47.4 | 41.105 | 29.625 |
| E1-W-T17-B1-1 | Groundwater | 2 | 13-14 | 4.97 | 70.4 | 69.1 | 27.4 |
| E1-W-T17-B2-1 | Groundwater | 2 | 13-14 | 5.17 | 53.7 | 35.32 | 34.59 |
| E1-W-T17-B3-1 | Groundwater | 2 | 13-14 | 4.96 | 65.8 | 58.075 | 26.855 |
| E1-W-T18-B1-1 | Groundwater | 3 | 13-14 | 4.77 | 82.6 | 32.045 | 29.26 |
| E1-W-T18-B2-1 | Groundwater | 3 | 13-14 | 4.88 | 86.8 | 23.98 | 30.765 |
| E1-W-T18-B3-1 | Groundwater | 3 | 13-14 | 4.99 | 86.7 | 31.94 | 27.24 |
| E1-W-T19-B1-1 | Groundwater | 1 | 13-14 | 4.86 | 70.4 | 39.62 | 26.985 |
| E1-W-T19-B2-1 | Groundwater | 1 | 13-14 | 4.96 | 70.7 | 23.295 | 27.92 |
| E1-W-T19-B3-1 | Groundwater | 1 | 13-14 | 4.98 | 72 | 25.165 | 27.93 |
| E1-W-T20-B1-1 | Groundwater | 2 | 13-14 | 4.8 | 72 | 61.54 | 28.145 |
| E1-W-T20-B2-1 | Groundwater | 2 | 13-14 | 4.98 | 66.5 | 20.68 | 27.23 |
| E1-W-T20-B3-1 | Groundwater | 2 | 13-14 | 5.07 | 69.5 | 22.7 | 27.535 |
| E1-W-T21-B1-1 | Groundwater | 3 | 13-14 | 4.78 | 63.8 | 64.765 | 29.2 |
| E1-W-T21-B2-1 | Groundwater | 3 | 13-14 | 5 | 66.1 | 24.17 | 28.355 |
| E1-W-T21-B3-1 | Groundwater | 3 | 13-14 | 4.97 | 84.1 | 41.435 | 26.515 |
| E1-W-T22-B1-1 | Groundwater | 1 | 13-14 | 4.88 | 77.2 | 68.485 | 30.38 |
| E1-W-T22-B2-1 | Groundwater | 1 | 13-14 | 4.98 | 73.8 | 34.175 | 28.44 |
| E1-W-T22-B3-1 | Groundwater | 1 | 13-14 | 5.04 | 71.9 | 30.34 | 25.935 |
| E1-W-T23-B1-1 | Groundwater | 2 | 13-14 | 4.95 | 72.1 | 62.315 | 27.675 |
| E1-W-T23-B2-1 | Groundwater | 2 | 13-14 | 5.08 | 66.7 | 32.875 | 33.46 |
| E1-W-T23-B3-1 | Groundwater | 2 | 13-14 | 4.93 | 80.7 | 32.845 | 26.425 |
| E1-W-T24-B1-1 | Groundwater | 3 | 13-14 | 4.6 | 67.1 | 64.285 | 26.61 |
| E1-W-T24-B2-1 | Groundwater | 3 | 13-14 | 4.89 | 67.5 | 35.715 | 26.55 |
| E1-W-T24-B3-1 | Groundwater | 3 | 13-14 | 4.92 | 68.5 | 32.44 | 26.65 |
| E2-R-T21-B1-1 | Rock |  | 18-19 | 4.1 | 62.4 | 66.865 | 25.02 |
| E2-R-T21-B1-2 | Rock |  | 18-19 | 4.1 | 62.4 | 66.865 | 25.02 |
| E2-R-T21-B1-3 | Rock |  | 18-19 | 4.1 | 62.4 | 66.865 | 25.02 |
| E2-R-T21-B2-1 | Rock |  | 18-19 | 4.14 | 58.8 | 67.665 | 22.48 |
| E2-R-T21-B2-2 | Rock |  | 18-19 | 4.14 | 58.8 | 67.665 | 22.48 |
| E2-R-T21-B2-3 | Rock |  | 18-19 | 4.14 | 58.8 | 67.665 | 22.48 |
| E2-R-T21-B3-1 | Rock |  | 18-19 | 4.24 | 63.5 | 67.825 | 22.895 |
| E2-R-T21-B3-2 | Rock |  | 18-19 | 4.24 | 63.5 | 67.825 | 22.895 |
| E2-R-T21-B3-3 | Rock |  | 18-19 | 4.24 | 63.5 | 67.825 | 22.895 |
| E2-R-T45-B1-1 | Rock |  | 18-19 | 4.38 | 70.3 | 62.46 | 17.635 |
| E2-R-T45-B1-2 | Rock |  | 18-19 | 4.38 | 70.3 | 62.46 | 17.635 |
| E2-R-T45-B1-3 | Rock |  | 18-19 | 4.38 | 70.3 | 62.46 | 17.635 |
| E2-R-T45-B2-1 | Rock |  | 18-19 | 4.46 | 65.6 | 59.55 | 16.06 |
| E2-R-T45-B2-2 | Rock |  | 18-19 | 4.46 | 65.6 | 59.55 | 16.06 |
| E2-R-T45-B2-3 | Rock |  | 18-19 | 4.46 | 65.6 | 59.55 | 16.06 |
| E2-R-T45-B3-1 | Rock |  | 18-19 | 4.42 | 60.9 | 52.96 | 17.275 |
| E2-R-T45-B3-2 | Rock |  | 18-19 | 4.42 | 60.9 | 52.96 | 17.275 |
| E2-R-T45-B3-3 | Rock |  | 18-19 | 4.42 | 60.9 | 52.96 | 17.275 |
| E2-W-T22-B3-1 | Groundwater |  | 18-19 | 4.32 | 79.3 | 69.68 | 22.015 |
| E2-W-T22-B2-1 | Groundwater |  | 18-19 | 4.24 | 80 | 68.07 | 21.32 |
| E2-W-T22-B1-1 | Groundwater |  | 18-19 | 4.17 | 82.3 | 73.835 | 22.59 |
| E2-W-T23-B3-1 | Groundwater |  | 18-19 | 4.38 | 58.2 | 45.265 | 21.19 |
| E2-W-T23-B2-1 | Groundwater |  | 18-19 | 4.33 | 55.9 | 45.585 | 21.085 |
| E2-W-T23-B1-1 | Groundwater |  | 18-19 | 4.3 | 57.8 | 43.72 | 20.41 |
| E2-W-T24-B3-1 | Groundwater |  | 18-19 | 4.37 | 62 | 48.765 | 20.26 |
| E2-W-T24-B2-1 | Groundwater |  | 18-19 | 4.41 | 53.3 | 45.625 | 20.245 |
| E2-W-T24-B1-1 | Groundwater |  | 18-19 | 4.29 | 56 | 49.675 | 21.585 |
| E2-W-T25-B3-1 | Groundwater |  | 18-19 | 4.37 | 61.3 | 51.155 | 20.48 |
| E2-W-T25-B2-1 | Groundwater |  | 18-19 | 4.51 | 56 | 41.875 | 20.48 |
| E2-W-T25-B1-1 | Groundwater |  | 18-19 | 4.37 | 62.8 | 49.24 | 20.295 |
| E2-W-T26-B3-1 | Groundwater |  | 18-19 | 4.39 | 58.7 | 48.29 | 19.86 |
| E2-W-T26-B2-1 | Groundwater |  | 18-19 | 4.37 | 56.1 | 44.255 | 19.975 |
| E2-W-T26-B1-1 | Groundwater |  | 18-19 | 4.35 | 60.3 | 50.23 | 20.84 |
| E2-W-T27-B3-1 | Groundwater |  | 18-19 | 4.39 | 58 | 50.01 | 20.345 |
| E2-W-T27-B2-1 | Groundwater |  | 18-19 | 4.38 | 59.4 | 47.675 | 20.24 |
| E2-W-T27-B1-1 | Groundwater |  | 18-19 | 4.37 | 60.3 | 54.85 | 20.095 |
| E2-W-T28-B3-1 | Groundwater |  | 18-19 | 4.38 | 60.9 | 48.835 | 19.74 |
| E2-W-T28-B2-1 | Groundwater |  | 18-19 | 4.48 | 53.3 | 30.845 | 20.51 |
| E2-W-T28-B1-1 | Groundwater |  | 18-19 | 4.3 | 58 | 51.06 | 20.33 |
| E2-W-T29-B3-1 | Groundwater |  | 18-19 | 4.4 | 60.7 | 46.4 | 20.205 |
| E2-W-T29-B2-1 | Groundwater |  | 18-19 | 4.42 | 58 | 46.595 | 19.615 |
| E2-W-T29-B1-1 | Groundwater |  | 18-19 | 4.42 | 56.9 | 48.74 | 20.605 |
| E2-W-T30-B3-1 | Groundwater |  | 18-19 | 4.38 | 62.8 | 48.41 | 19.515 |
| E2-W-T30-B2-1 | Groundwater |  | 18-19 | 4.37 | 55.1 | 46.38 | 19.685 |
| E2-W-T30-B1-1 | Groundwater |  | 18-19 | 4.33 | 56.3 | 50.845 | 19.67 |
| E2-W-T31-B3-1 | Groundwater |  | 18-19 | 4.43 | 57.6 | 49.04 | 19.345 |
| E2-W-T31-B2-1 | Groundwater |  | 18-19 | 4.37 | 56.3 | 51.21 | 21.345 |
| E2-W-T31-B1-1 | Groundwater |  | 18-19 | 4.33 | 56.9 | 49.485 | 19.48 |
| E2-W-T32-B3-1 | Groundwater |  | 18-19 | 4.39 | 53.5 | 48.075 | 19.135 |
| E2-W-T32-B2-1 | Groundwater |  | 18-19 | 4.39 | 55.3 | 47.94 | 19.41 |
| E2-W-T32-B1-1 | Groundwater |  | 18-19 | 4.37 | 56.9 | 48.155 | 19.44 |
| E2-W-T33-B3-1 | Groundwater |  | 18-19 | 4.46 | 56 | 45.745 | 19.53 |
| E2-W-T33-B2-1 | Groundwater |  | 18-19 | 4.69 | 48.9 | 15.735 | 19.32 |
| E2-W-T33-B1-1 | Groundwater |  | 18-19 | 4.37 | 51.6 | 47.075 | 19.035 |
| E2-W-T34-B3-1 | Groundwater |  | 18-19 | 4.5 | 54 | 41.615 | 18.515 |
| E2-W-T34-B2-1 | Groundwater |  | 18-19 | 4.82 | 51.5 | 15.1 | 19.985 |
| E2-W-T34-B1-1 | Groundwater |  | 18-19 | 4.48 | 51.6 | 41.36 | 19.005 |
| E2-W-T35-B3-1 | Groundwater |  | 18-19 | 4.56 | 60.5 | 38.015 | 18.805 |
| E2-W-T35-B2-1 | Groundwater |  | 18-19 | 4.52 | 59.8 | 42.45 | 18.98 |
| E2-W-T35-B1-1 | Groundwater |  | 18-19 | 4.53 | 61.3 | 34.815 | 18.45 |
| E2-W-T36-B3-1 | Groundwater |  | 18-19 | 4.55 | 58.8 | 41.875 | 18.09 |
| E2-W-T36-B2-1 | Groundwater |  | 18-19 | 4.54 | 56.5 | 40.175 | 18.055 |
| E2-W-T36-B1-1 | Groundwater |  | 18-19 | 4.5 | 64.1 | 44.55 | 19.03 |
| E2-W-T37-B3-1 | Groundwater |  | 18-19 | 4.52 | 53.6 | 42.885 | 18.04 |
| E2-W-T37-B2-1 | Groundwater |  | 18-19 | 4.5 | 50.5 | 42.67 | 17.845 |
| E2-W-T37-B1-1 | Groundwater |  | 18-19 | 4.49 | 56.5 | 45.28 | 18.685 |
| E2-W-T38-B3-1 | Groundwater |  | 18-19 | 4.48 | 60.3 | 53.03 | 18.115 |
| E2-W-T38-B2-1 | Groundwater |  | 18-19 | 4.47 | 57.3 | 50.26 | 17.765 |
| E2-W-T38-B1-1 | Groundwater |  | 18-19 | 4.89 | 47 | 8.41 | 18.55 |
| E2-W-T39-B3-1 | Groundwater |  | 18-19 | 4.43 | 66.5 | 55.905 | 17.72 |
| E2-W-T39-B2-1 | Groundwater |  | 18-19 | 4.65 | 57 | 27.96 | 17.79 |
| E2-W-T39-B1-1 | Groundwater |  | 18-19 | 4.45 | 58 | 58.3 | 18.11 |
| E2-W-T40-B3-1 | Groundwater |  | 18-19 | 4.4 | 63.3 | 59.405 | 17.235 |
| E2-W-T40-B2-1 | Groundwater |  | 18-19 | 4.43 | 63.1 | 56.795 | 17.815 |
| E2-W-T40-B1-1 | Groundwater |  | 18-19 | 4.42 | 61.4 | 63.995 | 18.555 |
| E2-W-T41-B3-1 | Groundwater |  | 18-19 | 4.48 | 61.8 | 55.785 | 17.085 |
| E2-W-T41-B2-1 | Groundwater |  | 18-19 | 4.41 | 67.7 | 58.405 | 17.27 |
| E2-W-T41-B1-1 | Groundwater |  | 18-19 | 4.32 | 62.1 | 59.59 | 18.445 |
| E2-W-T42-B3-1 | Groundwater |  | 18-19 | 4.42 | 60.8 | 56.15 | 18.065 |
| E2-W-T42-B2-1 | Groundwater |  | 18-19 | 4.41 | 58.9 | 58.44 | 16.62 |
| E2-W-T42-B1-1 | Groundwater |  | 18-19 | 4.26 | 59.9 | 61.165 | 20.9 |
| E2-W-T43-B3-1 | Groundwater |  | 18-19 | 4.4 | 65.9 | 58.75 | 17.28 |
| E2-W-T43-B2-1 | Groundwater |  | 18-19 | 4.42 | 60.8 | 54.3 | 18.24 |
| E2-W-T43-B1-1 | Groundwater |  | 18-19 | 4.36 | 60.9 | 56.16 | 17.89 |
| E2-W-T44-B3-1 | Groundwater |  | 18-19 | 4.47 | 66.3 | 57.245 | 17.31 |
| E2-W-T44-B2-1 | Groundwater |  | 18-19 | 4.41 | 65.9 | 57.98 | 16.805 |
| E2-W-T44-B1-1 | Groundwater |  | 18-19 | 4.29 | 65.3 | 60.86 | 18.12 |
| E2-W-T45-B3-1 | Groundwater |  | 18-19 | 4.42 | 60.9 | 52.96 | 17.275 |
| E2-W-T45-B2-1 | Groundwater |  | 18-19 | 4.46 | 65.6 | 59.55 | 16.06 |
| E2-W-T45-B1-1 | Groundwater |  | 18-19 | 4.38 | 70.3 | 62.46 | 17.635 |

**Supplementary Table 3**. P-values of the alpha diversity time series analysis’ parameters and the deviance explained by each model. Significant values are in bold.

|  | Bacteria *E1* | Bacteria *E2* | Eukaryote *E1* | Eukaryote *E2* |
| --- | --- | --- | --- | --- |
| DIC*DOC or DIC (p-value) | 0.99 | **9.10*10^-7^** | 0.15 | 0.94 |
| Time (p-value) | **0.0027** | **<2*10^-16^** | **<2*10^-16^** | **<2*10^-16^** |
| Time:B1 (p-value) | 0.52 | **2.93*10^-5^** | 0.83 | **7.69*10^4^** |
| Time:B2 (p-value) | 0.44 | 0.087 | 0.39 | **2.87*10^-5^** |
| Time:B3 (p-value) | 0.30 | 0.33 | **0.031** | **1.70*10^-5^** |
| Bioreactor, bs = ‘re’ (p-value) | 0.36 | **6.69*10^-5^** | **0.0055** | **9.59*10^-7^** |
| Deviance explained (%) | 37 | 94.2 | 62.2 | 94.9 |
| Variations between minimum and maximum | 0.6 | 1.25 | 1 | 1.25 |

**Supplementary Table 4**. Wilcoxon rank sum test comparing the evenness of sessile and planktonic communities for both the Bacteria and Eukaryote domains. Significant values are in bold.

|  | W | p-value |
| --- | --- | --- |
| Bacteria | 3123 | **0.02218** |
| Eukaryote | 4738 | **<2.2*10^-16^** |

**Supplementary Table 5.** Anova by contrast (999 permutations) on the Bray-Curtis dissimilarity index of each domain. Significant values are in bold. A) Bacteria; B) Eukaryote.

| A) |  |  |  |  |
| --- | --- | --- | --- | --- |
| Parameters | Df | SumOfSqs | F | Pr(>F) |
| Sample_typeRock | 1 | 6.132631 | 47.099015 | **0.001** |
| experienceE2 | 1 | 11.4686371 | 88.079899 | **0.001** |
| bioreactorB2 | 1 | 0.5819618 | 4.469505 | **0.002** |
| bioreactorB3 | 1 | 1.5382856 | 11.814136 | **0.001** |
| Sample_typeRock:experienceE2 | 1 | 2.3743153 | 18.2349 | **0.001** |
| Residual | 224 | 29.1664131 | NA | NA |

B)

| Parameters | Df | SumOfSqs | F | Pr(>F) |
| --- | --- | --- | --- | --- |
| Sample_typeRock | 1 | 4.323 | 23.7693 | **0.001** |
| experienceE2 | 1 | 20.069 | 110.3425 | **0.001** |
| bioreactorB2 | 1 | 0.495 | 2.7222 | **0.015** |
| bioreactorB3 | 1 | 0.859 | 4.7243 | **0.002** |
| Sample_typeRock:experienceE2 | 1 | 2.225 | 12.2356 | **0.001** |
| Residual | 224 | 40.74 |  |  |

**Supplementary Table 6**. Anova and variance partitioning results on the Bray-Curtis dissimilarity index of each community. The percentage of the variance explained by the bioreactors is for all bioreactors whereas the p-values are in contrast to B1. In addition to the values displayed in the table, the variance partitioning test could not disentangle the following effects: Bacteria *E1* shared 3% between bioreactor and DIC, and Eukaryote *E2* shared 2% between DIC and time. Significant values are in bold.

| Predictor variable | | Bacteria *E1* | | | Bacteria *E2* | | Eukaryote *E1* | | Eukaryote *E2* | |
| --- | --- | --- | --- | --- | --- | --- | --- | --- | --- | --- |
|  |  | Var. part (%) | p-value | | Var. part (%) | p-value | Var. part (%) | p-value | Var. part (%) | p-value |
| DIC | | 4 | | **0.001** | 3 | **0.001** | 6 | **0.001** | 8 | **0.001** |
| DOC | | 3 | | **0.001** | NA | NA | 7 | **0.001** | NA | NA |
| DIC:DOC | | NA | | 0.062 | NA | NA | NA | 0.274 | NA | NA |
| Time | | 15 | | **0.001** | 29 | **0.001** | 16 | **0.001** | 25 | **0.001** |
| Bioreactors | | 14 | |  | 8 |  | 4 |  | 20 |  |
|  | B2 |  | | **0.001** |  | **0.007** |  | 0.218 |  | **0.001** |
|  | B3 |  | | **0.001** |  | **0.001** |  | **0.001** |  | **0.001** |
